# Extra-electron induced covalent strengthening and generalization of intrinsic ductile-to-brittle criterion

**DOI:** 10.1038/srep00718

**Published:** 2012-10-09

**Authors:** Haiyang Niu, Xing-Qiu Chen, Peitao Liu, Weiwei Xing, Xiyue Cheng, Dianzhong Li, Yiyi Li

**Affiliations:** 1 Shenyang National Laboratory for Materials Science, Institute of Metal Research, Chinese Academy of Sciences, Shenyang 110016, China

## Abstract

Traditional strengthening ways, such as strain, precipitation, and solid-solution, come into effect by pinning the motion of dislocation. Here, through first-principles calculations we report on an extra-electron induced covalent strengthening mechanism, which alters chemical bonding upon the introduction of extra-valence electrons in the matrix of parent materials. It is responsible for the brittle and high-strength properties of Al_12_W-type compounds featured by the typical fivefold icosahedral cages, which are common for quasicrystals and bulk metallic glasses (BMGs). In combination with this mechanism, we generalize ductile-to-brittle criterion in a universal hyperbolic form by integrating the classical Pettifor's Cauchy pressure with Pugh's modulus ratio for a wide variety of materials with cubic lattices. This study provides compelling evidence to correlate Pugh's modulus ratio with hardness of materials and may have implication for understanding the intrinsic brittleness of quasicrystals and BMGs.

In the view of electronic structure, good ductile/plastic materials consisting of metallic elements are often characteristic of metallic bonding and the corresponding valence electrons are in a delocalized state^1^,^2^. For instance, pure aluminum (FCC Al) is usually soft and lacks strength since Al is regarded as a typical *s*, *p*-bonded metal nearly described by a classic free electron gas^3^. The high-strength brittle materials essentially consist of non-metal elements, i.e., the hardest diamond exhibits a strong directional and covalent bonding framework. Once the covalent bond is broken, new covalent bonds can not be easily and immediately reformed because of a high energy barrier. That's the reason that high-strength brittle materials often resist large stresses with little deformation and break without developing any plastic regime (namely, in a typically brittle nature). However, some ordered intermetallic compounds^4^ with periodic lattice structures, quasicrystals with ordered but not periodic atomic structures^5^ and BMGs with completely disordered atomic structures^6^, despite of metallic constituents in them, also exhibit essentially a common brittle nature at low temperature regime. In fact, it is an interesting issue to understand why those materials are intrinsically brittle although no non-metal elements (i.e., C, N, O, etc) participate in bonds.

Even good ductile/plastic materials can be strengthened by most traditional strengthening methods^7^, such as strain, solid-solution and dispersed precipitation^8^,^9^,^10^,^11^, which come into effect by impeding the motion of dislocation^12^,^13^. Here, through first-principles calculations, we highlighted a new type of strengthening way, extra-electron induced covalent strengthening, in the icosahedral Al_12_W-type intermetallic compounds^14^,^15^,^16^,^17^,^18^,^19^, Al_12_*X* (*X* = Cr, Mo, W, Mn, Tc and Re), which have attracted extensive interest^20^,^21^,^22^,^23^ since the quasicrystals^5^ are most related to the five-fold icosahedral structure. Although Al and transition metal elements *X* are both metallic, it has been found that, after introducing proper valence electrons in the center of the icosahedral cage of Al_12_, the electronic bonding feature is critically transformed into a covalent directional bonding framework from a free electron metallic bonding network, dramatically resulting in a brittle and hard nature in ordered Al_12_*X* intermetallic compounds. Certainly, this kind of extra-electron induced covalent strengthening is intrinsically different from traditional ways. Given the fact that icosahedral package is quite common for both quasicrytals^5^ and metal-metal-based metallic glasses^6^,^24^,^25^,^26^,^27^,^28^,^29^, this strengthening nature might shed light on the interpretation of their intrinsic brittleness. The analysis on the elastic properties revealed that the classic Pugh's modulus ratio (*G*/*B*)^30^ and Pettifor's Cauchy pressure (*C*_12_-*C*_44_)^31^ are well correlated with their ductile-to-brittle transition, also matching the metallic-to-covalent bonding transformation. Furthermore, we extend their correlation to a universal hyperbolic criterion to identify the ductile-to-brittle properties for as large as 332 materials with cubic lattice. Moreover, this unified criterion also provides evidence that Pugh's modulus ratio is closely correlated with hardness of materials as documented in our recently proposed model^32^,^33^ of hardness.

## Results

### Comparison of lattice structures between FCC Al and Al_12_X

**Figure 1:**
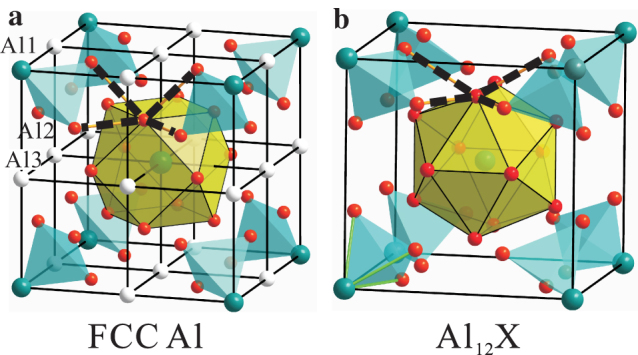
Comparison of the lattice structures between FCC Al and Al_12_*X*. (a), Supercell (2×2×2) of FCC Al.Here, Al atoms are classified into three types: Al1, Al2 and Al3. (b), Unit cell of Al_12_*X*. The small and large balls denote aluminum and *X* atoms, respectively. The Al_12_W-type structure is closely correlated with the FCC supercell when Al1 atom is replaced by a valence electron rich transition metal element *X* and the Al3 atoms have been removed.

A series of Al_12_*X* (*X* = Cr, Mo, W, Mn, Tc and Re) compounds^14^,^15^,^16^,^18^,^20^ crystallize in the Al_12_W-type structure with the space group of 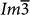 (No. 204) with *X* at the 2*a* site and with Al at the 24*g* site. As illustrated in Fig. 1, the Al_12_W-type structure is closely correlated with the FCC Al phase. In order to conveniently understand their correlation, Al atoms can be categorized into three classes Al1, Al2 and Al3 in the 2×2×2 FCC supercell (Fig. 1a). It has been noted that the Al1 and Al2 atoms correspond to the *X* and Al atoms in the Al_12_W-type structure, respectively (Fig. 1b). Their distinction lies in two aspects: (i) the Al_12_W-type structure lacks of Al3 atoms; (ii) in FCC Al each Al has twelve nearest-neighbor Al atoms to form an Al_12_ cuboctahedron whereas in the Al_12_W-type structure each *X* atom is surrounded by an icosahedron of twelve Al atoms (called Al_12_-icosahedron). In other words, the Al_12_-icosahedron can be considered as a distorted version of the Al_12_-cuboctahedron because of the removal of the Al3 atoms in FCC Al. The optimized structural parameters of Al_12_W-type compounds are in good agreement with available experimental data.

### Electronic structures and chemical bonds

**Figure 2:**
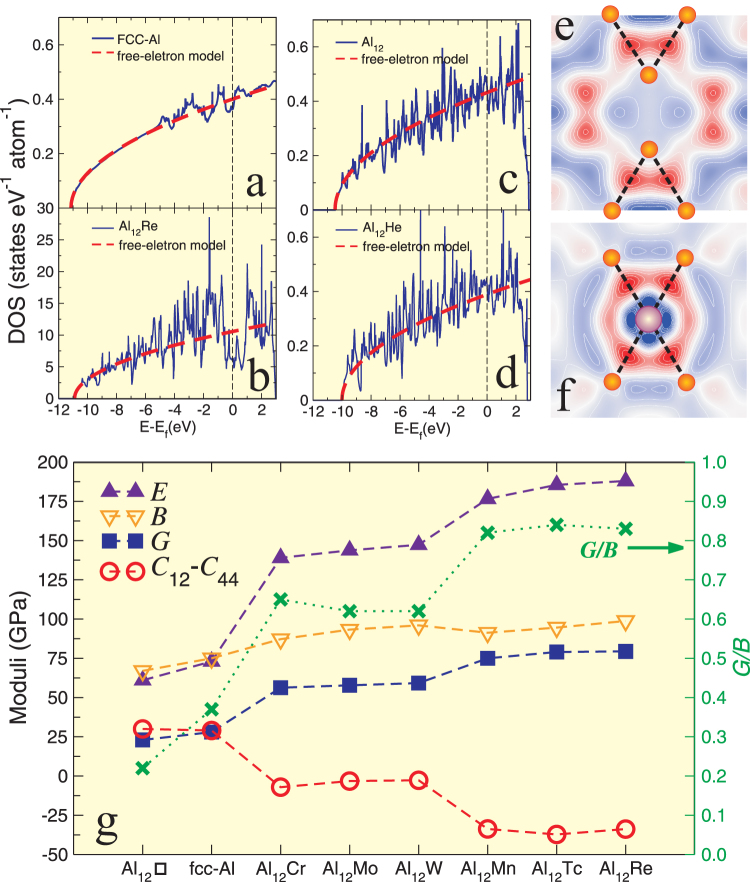
Extra-electron induced covalent strengthening. (a)-(d), Total DFT electronic densities of states of FCC-Al, Al_12_W-type Al_12_*X* (*X* = Re, □ and He). □ denotes that *X* is replaced by a vacancy. Here, Al_12_□ and Al_12_He are artificial and unstable, as evidenced by their positive enthalpies of formation (Supplementary Table S1). Their DOS profiles are compared with those obtained using the classic free electron model. e and f, Section contour maps of the difference of charge densities for e the Al-Al covalent bonds connecting the nearest-neighboring icosahedra as illustrated by the (020) plane and f the intra Al-Re covalent bonds within the Al_12_ icosahedron in the (0*y*0) plane of Al_12_Re. Similar results have been observed for all other Al_12_*X* (*X* = Cr, Mo, W, Mn and Tc), but are not shown here. The red and blue isovalues correspond to the charge accumulations and depletions, respectively. g, The comparison of calculated bulk moduli (*B* in GPa), Young moduli (*E* in GPa), shear moduli (*G* in GPa) and Cauchy pressure C_12_-C_44_ as well as Pugh'smodulus ratio of *G*/*B* (right side) in the series of Al_12_□, FCC Al, and Al_12_*X* (*X* = Cr, Mo, W, Mn, Tc and Re) (details refer to Supplementary Table S3 which summarizes all elastic data used here).

Although the FCC structure of Al is closely related to that of Al_12_W-type compounds, their electronic structures differ highly as evidenced in Fig. 2a and 2b. FCC Al exhibits a nearly free electron feature because its profile of the density of states can be described well through the classic free electronic theory. However, for Al_12_Re the appearance of a typical pseudogap at the Fermi level originated from the strong hybridization between Re *d*-orbital and Al *s*, *p*-orbitals^18^,^20^ evidences a significant deviation from the free electron feature. In fact, the similar pseudogap feature due to *sp-d* hybridization has been extensively observed in many other Al-based transition metal aluminides, such as Al_3_Ti and Al_3_V which were revealed to exhibit covalent bonding feature^34^,^35^.

In order to further understand the nature of the chemical bonding in Al_12_Re, we calculated the charge density differences of two planes as shown in Fig. 2e and 2f, (i) between two nearest neighboring Al_12_Re-icosahedra and (ii) between the sublattices of Re and Al_12_. The former is to check the inter-icosahedron bonding feature (corresponding to inter-Al-Al bonds as illustrated by a dashed line in Fig. 1b, whereas the latter is designed to show the intra-Al-Re bonding feature within each Al_12_Re icosahedron. It has been noted that apparent covalent Al-Al bonds can be confirmed due to the strong charge accumulations (*c.f.*, Fig. 2e). From Fig. 1b, each Al atom has four nearest neighbor Al atoms which are equivalently located in the two neighbor Al_12_-icosahedra. Interestingly, the similar covalent bonds between metallic atoms have been also reported not only in some intermetallic compounds (Al_3_Ti and Al_3_V)^34^,^35^ but also in the Re_2_C compound (Re-Re covalent bond^36^) according to Mulliken overlap population analysis as shown in Ref. [37]. In addition, Figure 2f compiles the charge density of the (020) plane which shows a directional Re-Al bond within each Al_12_-icosahedron. The significant charge accumulation along all Re-Al bonds can be visualized, representing their covalent feature. Re atoms occupy the center of each Al_12_-icosahedron and their nearest neighbors being twelve Al atoms are arranged in the form of a nearly perfect icosahedron. Hence, the twelve Re-Al covalent bonds are constructed in totally twelve different directions with a three-dimensional framework.

### Extra-electron induced covalent strengthening

Furthermore, we analyzed a series of other Al_12_*X* compounds as mentioned above. All of them exhibit a very similar electronic structure with the formation of a typical pseudogap (not shown here) and a covalent bonding framework. In terms of the chemical bonding nature of Al_12_W-type compounds, it would be naturally expected that they should have stronger mechanical properties than FCC Al due to the chemical bonding transformation from a metallic FCC-Al to a covalent Al_12_*X*. As expected, Fig. 2g evidences a dramatic increase of the elastic properties (in particular, shear (*G*) and Young (*E*) moduli) after the addition of *X*. Interestingly, it has been found that their mechanical performances are closely related to the valence electron number imposed by *X*. If no valence electrons are imposed by a vacancy (□) or an inert He atom, or a metallic element with a valence number smaller than 3 (i.e., Al) at *X*, their chemical bonding frameworks still remain metallic. From Fig. 2c for Al_12_□ and 2d for Al_12_He, their DOS profiles are similar to that of FCC Al. Although many peaks appear due to the reduced symmetry, the disappearance of the typical pesudogap is consistent with the lack of *sp-d* hybridization. In addition, it has been noted that their profiles can be further described by the classic free electron theory, evidencing the metallic bonding framework in those artificial compounds. Therefore, there is no doubt that their elastic properties (shear (*G*), Young (*E*) and bulk moduli (*B*)) are highly similar to those of FCC Al. However, when *X* is replaced by Cr, Mo, W, Mn, Tc, Re the valence electrons number of which exceeds that of Al, the abruptly increased mechanical properties are consistent with the formation of strong covalent bonding. From FCC Al (Al_12_□, Al_12_He, and Al_12_Al) to isoelectronic Al_12_*X* (*X* = Cr, Mo and W), their *E* and *G* are abruptly increased by more than 100%. These values are even increased much heavier in the series of isoelectronic Al_12_*X* (*X* = Mn, Tc, Re) which have one more valence electron introduced in each Al_12_-icosahedron than the cases of *X* = Cr, Mo and W. Hence, we have defined this feature from metallic bonding to covalent bonding transformation in combination with the mechanical strengthening depending on the introduction of valence electrons of a critical number larger than three imposed by *X* in Al_12_*X* as an extra-electron induced covalent strengthening mechanism.

### A universal ductile-to-brittle criterion

**Figure 3:**
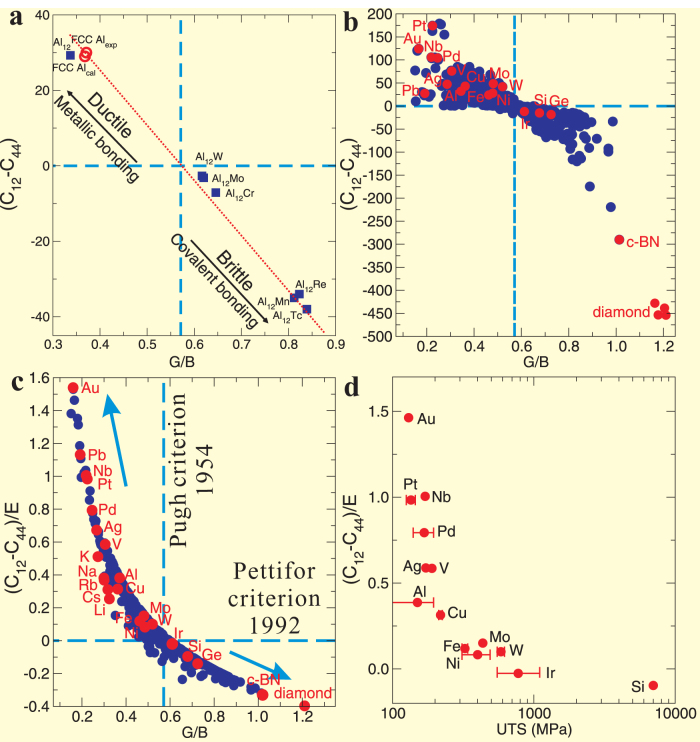
A universal ductile-to-brittle criterion. (a), Nearly linear correlation between *C*_12_-*C*_44_ and *G*/*B* for those Al_12_X aluminides. (b), The correlation in a is further extended to a large scale data collected for 332 compounds (571 group data sets; Supplementary Tables S2 and S3) from literature. (c), A renormalized hyperbolic correlation derived by dividing Young modulus *E* from (C_12_-C_44_) for all the summarized data of b. The horizontal line of C_12_-C_44_ denotes the critical zero Cauchy pressure defined by Pettifor^31^, whereas the vertical line of *G*/*B* = 0.571 corresponds to critical Pugh's modulus ratio defined by Pugh^30^. (d), The relation between Cauchy pressure and the experimental ultimate intensile strength (UTS)^38^ for selected solid phases with cubic lattice of some pure elements. For details, see text.

Interestingly, this mechanism is further supported by both classical criteria of Cauchy pressure *C*_12_-C_44_ (as proposed by Pettifor in 1992^31^) and of Pugh's modulus ratio *G*/*B* (as proposed by Pugh^30^). From Fig. 2g, for both FCC Al and Al_12_□ their *C*_12_-C_44_ remains nearly the same positive value, implying the metallic bonding framework in terms of Pettifor's suggestion^31^. In addition, their *G*/*B* values also meet Pugh's criterion when *G*/*B* is smaller than 0.571, in agreement with a ductile property. However, when *X* is replaced by transition metal elements (*X* = Cr, Mo, W, Mn, Tc, and Re) their *C*_12_-C_44_ values are all positive, revealing a directional (covalent) bonding framework from Pettifor's criterion of Cauchy pressure and their *G*/*B* values are all larger than 0.571, suggesting their brittle mechanical properties based on Pugh's criterion of modulus ratio. In order to further assess the influence of the Pugh's modulus ratio (*G*/*B*) and Cauchy pressure (*C*_12_-*C*_44_) on the mechanical properties of cubic materials, we also plotted in Fig. 3a *C*_12_-*C*_44_ against *G*/*B* for these Al_12_X. Unexpectedly, Figure 3a shows a nearly linear relationship, building a nice connection between the classic Pugh's modulus ratio and the classic Cauchy pressure. Because the artificial compound of Al_12_□ shows an electronic structure and metallic bonding feature similar to FCC Al, we see that it locates closest to FCC Al in the upper left corner. However, Al_12_*X* compounds are dramatically moved to the lower right corner mainly due to the presence of directional covalent bonding, resulting in a brittle behavior. In particular, Al_12_*X* (*X* = Mn, Tc and Re) locates in the lower right corner. In this sense, there is no doubt that they should be the most brittle and strongest materials among those Al_12_X aluminides collected here.

When a wide variety of samples (in total, 571 group data sets for 332 compounds collected from literature) are included for comparison, their correlation does not remain linearly any more, rather revealed a highly scattered distribution in Fig. 3b. Strikingly, when Cauchy pressure C_12_-C_44_ is renormalized by multiplying with a factor of 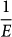 (*E* – Young modulus), all those data in Fig. 3b can be, unexpectedly, uniformed as a beautiful hyperbola (Fig. 3c). The most spectacular fact is that diamond, the hardest known of highest strength material, locates at the lowest right corner with the largest *G*/*B* ratio and the lowest 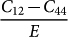. In contrast, the most ductile and plastic Au exhibits the most positive (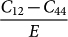) and the lowest Pugh's modulus ratio. This fact demonstrated that this hyperbolic relationship can be unified as a rule to identify the intrinsic strength and ductility of cubic materials. If we use Pugh's modulus *G*/*B* as a factor of strength, as demonstrated by ultimate tensile strength (UTS) experimentally measured for some pure elemental solids with cubic lattice in Fig. 3d, and the revised Cauchy pressure 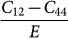 as a factor of ductility, the hyperbolic correlation exactly shows a well-known fact for materials^7^,^8^. Namely, the high-strength materials, in general, lack of good ductility, whereas the ductile materials do not exhibit good strength.

### The application of ductile-to-brittle criterion to hardness of materials

**Figure 4:**
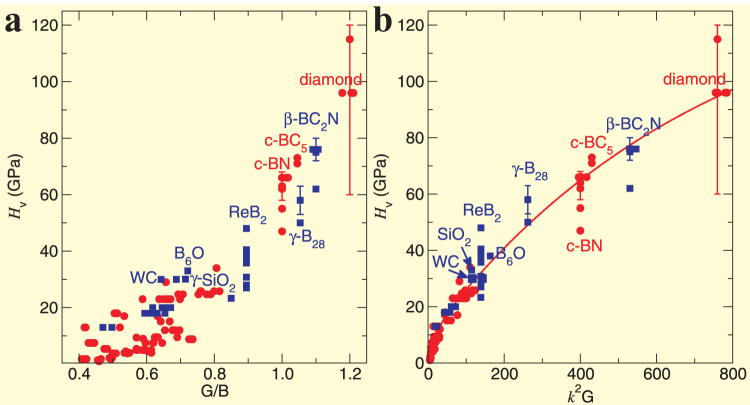
The application of ductile-to-brittle criterion to hardness of materials. (a), Correlation between Experimental Vickers hardness (H*_v_*) and its Pugh's modulus ratio (*k* = *G*/*B*) for hard materials. (b), Experimental Vickers hardness as a function of a product (*k*^2^*G*) between the squared Pugh's modulus ratio (*k*^2^) and shear modulus *G*^33^,^34^. Circles correspond to hard materials with cubic structure, whereas solid squares denote non-cubic-lattice hard materials (Supplementary Table S4).

From Fig. 3c it can be seen that Pugh's modulus ratio (*k* = *G*/*B*) seems to mirror the hardness of materials. For instance, the hardest diamond has a largest *k* of 1.2 and the second hardest cubic-BN has a *k* of about 1.0, just smaller than that of diamond. Therefore, it is our aim to further check a wide variety of hard materials by comparing their experimental Vickers hardness (*H_v_*) as a function of *k* in Fig. 4a. The resulting trend seems to show a good correlation, namely, hardness increases with increasing *k*, despite of some scattering data available. Given this fact that the Poisson's ratio^39^ (*ν*) is reversely proportional to *k*, accordingly, hardness certainly exhibits a decreasing tendency as *ν* increases. This fact provides the compelling evidence to validate our recently proposed hardness model^32^,^33^, *H_v_* = 2(*k*^2^*G*)^0.585^−3. It indicates that the hardness not only correlates with shear modulus as observed by Teter^40^, but also with bulk modulus as observed by Gilman^41^. Our work combines those aspects^32^ that were previously argued strongly, and, most importantly, is capable to correctly reproduce the hardness of a wide variety of hard materials including all known superhard materials as illustrated in Fig. 4b.

## Discussion

We have systematically investigated a series of Al_12_*X* (*X* = Cr, Mo, W, Mn, Tc and Re) intermetallic compounds, revealing their brittle and high-strength mechanical properties. This class of compounds attracts our interest because their structures are characteristic of the ordered icosahedral units which are composed of Al_12_ cages with a centered transition metal *X*. This unique five-fold icosahedral feature is similar to the basic structural unit of some typical quasicrystals and BMGs. Interestingly, we have established a direct structural connection between Al_12_*X* and FCC Al, and the latter is, apparently, a typical metal whose electronic structure can be described well within free-electron gas model. We found that the introduction of extra-valence electrons (typically, larger than three) imposed by *X* induces a covalent bonding framework in Al_12_*X*. These covalent bonds are not only for all the intra-Al-*X* bonds within each icosahedron but also for the inter-Al-Al bonds between any two neighboring icosahedra. Undoubtedly, the occurrence of the covalent bonding framework results in the typical brittle and high-strength properties of Al_12_*X*. We have then proposed a new strengthening way, called extra-electron induced covalent strengthening. Our findings extend the classical strengthening ways of metal from a mechanical viewpoint (typically, pinning dislocations by solid-solution, precipitation and stress) to an electronic viewpoint by modifying chemical bonds in the local or whole materials. We believe that the extra-electron induced covalent strengthening may originally exist in materials but perhaps, was neglected in some classical ways because one used to focus on dislocation effect from mechanical aspects. Therefore, we cannot rule out the possibility that, in some solid-solution strengthening cases, the solution addition may have an effect on modifying the local chemical bonds which lift up the activation energy barrier of dislocations, thereby taking action of strengthening effect. In addition, this new way would also have some potential applications to accomplish the whole or local strengthening effects (i.e., on the surface strengthening) through the introduction of extra electrons provided by proper alloying addition.

From the ductile FCC Al to the brittle and high-strength Al_12_X, the transition of electronic chemical bonding from metallic to covalent framework is consistent with the enhanced elastic mechanical properties interpreted well by empirical Pettifor's criterion of Cauchy pressure (*C*_12_-*C*_44_)^31^ and Pugh's modulus ratio (*G*/*B*)^30^. As mentioned above, Pettifor highlighted a critical zero Cauchy pressure (*C*_12_-*C*_44_) to separate the metallic and covalent (directional) bonding framework and Pugh also yielded a critical value of *G*/*B* = 0.571 as a boundary between ductile and brittle properties. We have shown the interplay between these two empirical criteria proposed by Pettifor and Pugh can be unified as an intrinsic ductile-to-brittle criterion in a universal hyperbolic correlation (see Fig. 3c), by fitting a wide range of materials with cubic lattices collected from 332 compounds in the total 557 group data set. As a consequence of the generalization of their criteria, this new form of the hyperbolic relation indeed uncovers there is no so-called critical separated boundaries between the ductile and brittle properties of materials. In particular, as shown in Fig. 3c, the crossing point between the hyperbolic curve and the vertical dashed line of the critical value (*G*/*B* = 0.571) defined by Pugh^30^ exactly corresponds to the critical zero value of *C*_12_-*C*_44_ proposed by Pettifor^31^. This fact evidences the intrinsic coherency of these two classical criteria. It still needs to be emphasized that, although mechanical properties related with permanent deformation of materials are highly complex, the currently unified criterion seems to provide the simple and start-up applications to identify roughly the intrinsic ductile-to-brittle property. Given the fact that our collected data for those 332 compounds all correspond to room temperature for experiments and to sufficiently low temperature (i.e., absolute zero) for *ab initio* calculations, this empirical correlation should be thus limited to the low-temperature scale of materials. In addition, this unified criterion also unveil the substantial evidence that Vickers hardness of materials is correlated with Pugh's modulus ratio^32^,^33^, as successfully applied to CrB_4_^42^ and WB_3_/WB_4_^43^ as well as a series of superhard phases of cold-compressed graphite^44^.

## Methods

First-principles calculations were performed using the Vienna *ab initio* Simulation Package (VASP)^45^ with the ion-electron interaction described by the projector augmented wavepotential (PAW)^46^. The energy cutoff for the plane-wave expansion of eigenfunctions was set to 500 eV. We used the generalized gradient approximation (GGA) based on the Perdew-Burke-Ernzerhof (PBE) scheme^47^ for the exchange-correlation functional. Optimization of structural parameters was achieved by minimizing forces and stress tensors. Highly converged results were obtained utilizing a dense 13×13×13 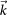-point grid for the Brillouin zone integration. The independent elastic constants of the Al_12_W-type icosahedral compounds were derived from the total energies as a function of lattice strains^48^. These strain energies were fitted to third-order polynomials from which the elastic constants at the equilibrium structures were calculated.

## Author Contributions

X.-Q.C., D. Z. L. and Y. Y. L. designed and coordinated the overall study and X.-Q.C. wrote the paper. H.Y.N. and X.-Q.C. performed theoretical calculations and H.Y.N. collected all data with the help from P.T.L., W.W.X. and X. Y. C. All contributed to the discussion of the results.

## Supplementary Material

Supplementary Information Online supplementary Materials
