# Supplementary material for: Extra-electron induced covalent strengthening and generalization of intrinsic ductile-to-brittle criterion
Source: Sci Rep. 2012 Oct 9;2:718. doi: 10.1038/srep00718 (PMC3466921; doi:10.1038/srep00718)
Supplement: Supplementary Information — Online supplementary Materials [file srep00718-s1.pdf]

Online Supporting Materials

Extra-electron induced covalent strengthening and generation of intrinsic ductile-to-brittle criterion

HaiyangNiu, Xing-Qiu Chen*, Peitao Liu, Weiwei Xing, Xiyue Cheng, Dianzhong Li, andYiyi Li

Shenyang National Laboratory for Materials Science, Institute of Metal Research, Chinese Academy of Sciences, Shenyang, 110016, P. R. China

(**Corresponding author: xingqiu.chen@imr.ac.cn*)

Content page

1. Table S1 2
2. Table S23
3. Table S322
4. Table S4 25
5. References 29

Table s1: Crystal lattice constants (*a* in Å) and enthalpy of formation (ΔH in eV·atom^{-1}) of Al_{12}-W-type compounds and FCC Al.

compounds	*a* (Å)		ΔH (eV·atom^{-1})	
	DFT	EXPT	DFT	EXPT
$Al_{12}He$	7.622		0.179	
Al_{12}	7.550		0.095	
$Al_{12}Al$	7.654		0.063	
Al	4.046	4.045[s1]	0	
$Al_{12}Cr$	7.506	7.507[s2]	-0.071	
$Al_{12}Mo$	7.582	7.573[s2]	-0.117	-0.16[s6]
$Al_{12}W$	7.587	7.580[s2]	-0.079	
$Al_{12}Mn$	7.469	7.470[s3]	-0.115	
$Al_{12}Tc$	7.534	7.528[s4]	-0.168	
$Al_{12}Re$	7.537	7.532[s5]	-0.137	

Table s2 The theoretical and available experimental elastic constants C_{ij} (GPa), shear G (GPa), bulk B (GPa), Young E (GPa) moduli and Poisson ratio v, Pugh's modulus ratio G/B, Cauchy Pressure C_{12}-C_{44} (CP), (C_{12}-C_{44})/E for a series of compounds with cubic lattices. In the last column, "e" and "c" denotes elastic constants from direct experimental measured and theoretical calculations, respectively.

No.	Compounds	Refs	C_{11}	C_{12}	C_{44}	G	B	E	v	G/B	CP/E	
1	PdZr	s31	152.90	141.30	34.10	17.17	145.17	49.35	0.44	0.12	2.17	c
2	$CuAuZn_2$	s8	136.00	130.00	52.90	19.93	132.00	55.81	0.43	0.15	1.38	e
3	KCN	s9	19.40	11.80	1.50	2.20	14.33	6.27	0.43	0.15	1.64	e
4	AgCl	s8	59.60	36.10	6.22	8.05	43.93	22.75	0.41	0.18	1.31	e
5	AgBr	s9	59.20	36.40	6.16	7.90	44.00	22.36	0.42	0.18	1.35	e
		s8	56.30	32.80	7.26	8.81	40.63	24.66	0.40	0.22	1.04	e
6	AuZn	s8	141.80	126.30	54.50	25.88	131.47	72.25	0.41	0.20	0.99	e
7	$AuGa_2$	s8	100.20	73.40	29.90	21.67	82.33	59.73	0.38	0.26	0.73	e
8	Ir_4C	s32	530.00	212.00	57.00	87.24	318.00	239.52	0.37	0.27	0.65	c
9	CeAg	s8	59.80	45.20	21.80	14.07	50.07	38.55	0.37	0.28	0.61	e
10	Cu_3Au	s8	191.00	138.00	66.30	45.90	155.67	125.28	0.37	0.29	0.57	e
		s8	185.00	135.00	68.70	45.83	151.67	124.75	0.36	0.30	0.53	e
		s8	187.00	135.00	67.80	46.17	152.33	125.69	0.36	0.30	0.53	e
		s8	177.00	134.00	65.50	41.96	148.33	114.85	0.37	0.28	0.60	e
11	$Pb(NO_3)_2$	s9	37.29	27.65	13.47	8.93	30.86	24.39	0.37	0.29	0.58	e
12	LaAg	s8	60.30	43.20	20.90	14.60	48.90	39.82	0.36	0.30	0.56	e
13	CdS	s8	77.90	52.70	24.10	18.58	61.10	50.59	0.36	0.30	0.57	e
14	CeTe	s8	129.00	26.00	7.25	18.00	60.33	48.51	0.37	0.30	0.39	e

15	HeSe	s8	60.80	44.60	22.30	14.86	50.00	40.53	0.36	0.30	0.55	e
16	Nb_3Sn	s8	255.00	114.00	40.10	50.36	161.00	136.80	0.36	0.31	0.54	e
17	CdF_2	s8	184.00	67.00	21.80	32.79	106.00	89.08	0.36	0.31	0.51	e
18	CdSe	s8	66.70	46.30	22.30	16.29	53.10	44.32	0.36	0.31	0.54	e
19	$ZnGa_2O_4$	s33	287.70	218.30	124.90	75.03	241.43	203.37	0.36	0.31	0.46	c
		s33	390.41	142.77	113.77	117.69	225.32	300.71	0.28	0.52	0.10	c
20	$AlCu_2Zr$	s14	157.50	115.31	62.69	40.55	129.37	109.97	0.36	0.31	0.48	c
21	$AlCu_3$	s13	176.00	117.40	92.40	58.40	136.93	153.02	0.31	0.43	0.16	c
		s14	150.71	120.57	81.88	42.34	130.61	113.71	0.35	0.32	0.34	c
22	Cu_3N	s34	234.20	54.40	17.50	36.14	114.33	97.39	0.36	0.32	0.38	c
23	$HoCo_2$	s8	153.00	93.00	40.10	35.70	113.00	96.90	0.36	0.32	0.55	e
24	Cu_4N	s34	230.50	95.70	36.10	46.48	140.63	125.57	0.35	0.33	0.47	c
25	$CePb_3$-200k	s8	78.50	53.70	28.60	20.45	61.97	55.24	0.35	0.33	0.45	e
26	CdTe	s8	53.50	36.90	20.20	14.14	42.43	38.15	0.35	0.33	0.44	e
27	CaTe	s9	53.51	36.81	19.94	14.06	42.38	37.96	0.35	0.33	0.44	e
28	HgTe	s9	54.80	38.10	20.40	14.26	43.67	38.54	0.35	0.33	0.46	e
29	$Sr(NO_3)_2$	s9	42.55	29.21	15.90	11.22	33.66	30.28	0.35	0.33	0.44	e
30	$LaCRh_3$	s35	281.00	145.00	63.00	64.95	190.33	174.96	0.35	0.34	0.47	c
		s35	258.00	139.00	74.00	67.81	178.67	180.59	0.33	0.38	0.36	c
31	$ZnCNi_3$	s36	319.53	105.72	39.42	59.58	176.99	160.50	0.35	0.34	0.41	c
		s36	344.00	110.00	64.00	81.68	188.00	214.00	0.31	0.43	0.21	c
32	LiIn	s8	55.90	41.70	26.70	15.77	46.43	42.36	0.35	0.34	0.35	e
33	HeTe	s8	53.20	36.80	20.80	14.32	42.27	38.57	0.35	0.34	0.41	e
34	Y_2O_3	s30	213.60	112.90	72.60	62.70	146.47	164.60	0.31	0.43	0.24	c
		s30	241.90	128.00	85.10	72.45	165.97	189.72	0.31	0.44	0.23	c
		s30	224.00	112.00	74.60	66.51	149.33	173.73	0.31	0.45	0.22	e
		s8	227.00	138.00	68.60	57.67	167.67	155.21	0.35	0.34	0.45	e
35	Pd_4C	s32	238.00	158.00	95.00	67.15	184.67	179.53	0.34	0.36	0.35	c

36	$MgCNi_3$	s36	342.40	81.40	44.50	69.66	168.40	183.32	0.32	0.41	0.20	c
		s36	340.00	102.00	57.00	76.90	181.33	202.05	0.31	0.42	0.22	c
		s36	309.47	101.84	42.64	61.45	171.05	164.50	0.34	0.36	0.36	c
37	CdO	s37	183.99	96.01	45.78	45.06	125.34	120.70	0.34	0.36	0.42	c
38	UCo_2	s8	219.00	127.00	65.40	56.80	157.67	152.13	0.34	0.36	0.40	e
39	PbF_2	s9	88.80	47.20	24.54	22.97	61.07	61.23	0.33	0.38	0.37	e
		s8	93.00	46.00	21.30	22.15	61.67	59.36	0.34	0.36	0.42	e
40	ZnO	s8	204.00	113.00	54.90	50.92	143.33	136.60	0.34	0.36	0.43	e
41	$CoZnFeO_2$	s9	266.00	153.00	78.00	68.55	190.67	183.63	0.34	0.36	0.41	e
42	$CdCNi_3$	s36	311.46	126.73	57.23	69.39	188.31	185.39	0.34	0.37	0.37	c
		s36	341.00	127.00	72.00	84.42	198.33	221.78	0.31	0.43	0.25	c
		s36	255.00	101.65	58.39	65.13	152.77	171.07	0.31	0.43	0.25	c
43	$TlCY_3$	s38	169.70	63.10	27.90	36.27	98.63	96.89	0.34	0.37	0.36	c
44	NbN	s39	739.00	161.00	76.00	134.49	353.67	356.66	0.33	0.38	0.24	c
		s39	608.00	134.00	117.00	155.86	292.00	396.77	0.27	0.53	4.28	e
45	$MgCu_2$	s40	125.00	71.70	42.30	35.15	89.47	93.23	0.33	0.39	0.32	c
		s8	123.00	70.60	41.20	34.36	88.07	91.21	0.33	0.39	0.32	e
46	$PrPb_3$	s8	66.80	43.00	27.70	19.74	50.93	52.40	0.33	0.39	0.29	e
47	CsF	s8	44.20	15.40	7.58	9.83	25.00	26.07	0.33	0.39	0.30	e
48	CoO	s9	261.23	147.00	83.00	71.45	185.08	189.92	0.33	0.39	0.34	e
		s8	260.00	145.00	82.40	71.34	183.33	189.44	0.33	0.39	0.33	e
49	TlCl	s8	40.30	15.50	7.69	9.32	23.77	24.73	0.33	0.39	0.32	e
50	YbB_6	s8	335.00	81.00	40.00	64.95	165.67	171.92	0.33	0.39	0.24	e
51	$LiCu_3N$	s 34	196.70	57.60	29.30	41.76	103.97	110.41	0.32	0.40	0.26	c
52	$TcCNi_3$	s 36	357.00	148.00	76.00	86.35	217.67	228.80	0.32	0.40	0.31	c
53	$InCY_3$	s 38	173.20	64.00	33.30	40.64	100.40	107.41	0.32	0.40	0.29	c
54	$SrCl_2$	s 8	70.50	19.00	9.60	14.44	36.17	38.18	0.32	0.40	0.25	e
55	TlBr	s 8	37.60	14.80	7.54	8.90	22.40	23.58	0.32	0.40	0.31	e

		s 9	37.60	14.58	7.57	8.96	22.25	23.69	0.32	0.40	0.30	e
56	$LuCRh_3$	s35	354.00	150.00	81.00	88.83	218.00	234.63	0.32	0.41	0.29	c
57	$YCRh_3$	s35	337.00	146.00	79.00	85.23	209.67	225.18	0.32	0.41	0.30	c
58	$AlNi_3$	s11	278.00	212.00	186.00	94.98	234.00	248.38	0.32	0.41	0.10	c
		s8	223.50	149.00	122.90	76.33	173.83	199.17	0.31	0.44	0.13	e
		s12	225.00	149.00	124.00	77.34	174.33	201.56	0.31	0.44	0.12	c
		s11	221.00	146.00	124.00	76.95	171.00	200.14	0.30	0.45	0.11	e
		s8	223.00	148.00	125.00	77.33	173.00	201.29	0.31	0.45	0.11	e
		s8	220.60	146.10	124.00	76.75	170.93	199.67	0.31	0.45	0.11	e
		s12	242.00	152.00	125.00	83.03	182.00	215.84	0.30	0.46	0.13	c
59	Ni_3Ga	s11	191.00	123.00	109.00	68.45	145.67	177.05	0.30	0.47	0.08	e
		s11	296.00	226.00	202.00	102.33	249.33	267.08	0.32	0.41	0.09	c
60	GdCu	s8	86.80	40.60	23.10	23.10	56.00	60.92	0.32	0.41	0.29	e
61	Sc_2O_3	s8	290.00	151.00	89.00	80.61	197.33	212.85	0.32	0.41	0.29	e
62	UO_2	s9	396.00	121.00	64.10	87.48	212.67	230.68	0.32	0.41	0.25	e
		s8	396.00	121.00	64.10	87.48	212.67	230.68	0.32	0.41	0.25	e
63	c-ZnS	s8	102.00	64.60	44.60	31.47	77.07	83.03	0.32	0.41	0.24	e
		s9	104.62	65.34	46.13	32.75	78.43	86.18	0.32	0.42	0.22	e
		s8	116.60	71.80	50.90	36.62	86.73	96.23	0.32	0.42	0.22	e
		s30	96.50	56.50	44.90	32.46	69.83	84.25	0.30	0.46	0.14	c
		s9	80.96	48.81	44.05	29.42	59.53	75.64	0.29	0.49	0.06	e
64	AlNi	s8	212.00	143.00	112.00	70.00	166.00	183.62	0.32	0.42	0.17	e
		s15	199.00	137.00	116.00	68.66	157.67	179.12	0.31	0.44	0.12	e
		s15	208.20	132.60	112.70	72.80	157.80	188.89	0.30	0.46	0.11	c
		s16	204.60	135.40	116.80	71.90	158.47	186.78	0.30	0.45	0.10	c
		s15	200.00	140.00	120.00	69.27	160.00	180.70	0.31	0.43	0.11	c
65	Rh4C	s32	463.00	170.00	95.00	113.07	267.67	297.33	0.31	0.42	0.25	c
66	$ScCRh_3$	s35	390.00	146.00	81.00	95.49	227.33	251.27	0.32	0.42	0.26	c

		s35	367.00	146.00	81.00	91.74	219.67	241.59	0.32	0.42	0.27	c
67	$AgCNi_3$	s36	312.00	104.00	57.00	72.69	173.33	191.28	0.32	0.42	0.25	c
68	ZrO_2	s41	499.00	111.00	63.00	100.86	240.33	264.85	0.32	0.42	0.18	c
		s41	417.00	82.00	47.00	80.59	193.67	211.58	0.32	0.42	0.17	e
		s41	455.00	64.00	63.00	101.22	194.33	258.03	0.28	0.52	0.00	c
69	CoPt	s8	290.00	178.00	124.00	90.13	215.33	237.15	0.32	0.42	0.23	e
70	TmAg-200K	s8	112.00	62.00	40.00	33.13	78.67	87.15	0.32	0.42	0.25	e
71	$CdAl_2O_4$	s42	292.00	134.00	78.00	78.40	186.67	206.31	0.32	0.42	0.27	c
		s42	231.00	149.00	115.00	76.09	176.33	199.24	0.31	0.43	0.17	c
72	$SnCMn_3$	s43	544.06	138.52	80.84	118.03	273.70	309.25	0.31	0.43	0.19	c
		s43	476.66	118.66	73.35	105.81	237.99	276.20	0.31	0.44	0.16	c
73	FeTi	s8	325.00	121.00	69.00	80.73	189.00	211.99	0.31	0.43	0.25	e
		s8	310.00	86.00	74.90	88.04	160.67	223.32	0.27	0.55	0.05	e
74	MnO	s9	223.00	120.00	79.00	66.55	154.33	174.55	0.31	0.43	0.23	e
		s8	227.00	116.00	78.00	68.06	153.00	177.81	0.31	0.44	0.21	e
75	Ni_3Fe	s8	236.10	150.30	119.40	79.25	178.90	206.81	0.31	0.44	0.15	e
76	AgMg	s8	83.80	56.40	47.60	28.98	65.53	75.51	0.31	0.44	0.12	e
		s8	84.60	56.70	48.50	29.52	66.00	76.81	0.31	0.45	0.11	e
77	BaF_2	s8	91.10	41.20	25.30	25.16	57.83	65.92	0.31	0.44	0.24	e
		s9	91.99	41.57	25.68	25.49	58.38	66.76	0.31	0.44	0.24	e
78	$KZnF_3$	s44	111.75	49.76	31.37	31.22	70.43	81.61	0.31	0.44	0.23	c
		s44	170.72	63.54	45.05	48.29	99.27	124.65	0.29	0.49	0.15	c
		s44	134.50	52.70	38.10	39.20	79.97	101.07	0.29	0.49	0.14	c
79	Ni_3Mn	s11	245.00	165.00	145.00	86.87	191.67	225.50	0.30	0.45	0.09	e
80	Ni_3Ge	s11	263.00	143.00	103.00	82.93	183.00	216.10	0.30	0.45	0.19	e
		s11	326.00	200.00	190.00	122.19	242.00	313.02	0.28	0.50	0.03	c
81	$ZrCNi_3$	s36	312.00	110.00	69.00	80.41	177.33	209.54	0.30	0.45	0.20	c
82	YAg	s45	102.40	54.00	37.20	31.31	70.13	81.76	0.31	0.45	0.21	e

		s45	105.00	50.00	37.00	32.85	68.33	84.95	0.29	0.48	0.15	c
83	RbI	s9	25.56	3.82	2.78	4.99	11.07	12.94	0.31	0.45	0.08	e
		s8	25.60	3.70	2.79	5.01	11.00	12.99	0.30	0.46	0.07	e
84	TbCu	s46	122.81	51.34	32.37	33.68	75.16	87.90	0.31	0.45	0.22	c
		s46	146.53	52.93	33.74	38.47	84.13	100.15	0.30	0.46	0.19	c
85	$CaHfO_3$	s47	450.74	62.94	49.76	89.12	192.21	230.43	0.30	0.46	0.06	c
86	YCu	s45	113.40	48.40	32.30	32.38	70.07	84.17	0.30	0.46	0.19	e
		s45	116.00	47.00	35.00	34.80	70.00	89.56	0.29	0.50	0.13	c
87	Cu_2MnAl	s8	135.00	97.00	94.00	50.22	109.67	129.59	0.30	0.46	0.02	e
88	Ce_3S_4	s8	111.60	46.80	31.40	31.80	68.40	82.59	0.30	0.46	0.19	e
89	RbF	s8	55.20	14.00	9.25	12.83	27.73	33.32	0.30	0.46	0.14	e
90	V_3Ge	s8	294.00	107.00	69.90	78.54	169.33	204.08	0.30	0.46	0.18	e
91	ZnSe	s8	86.40	51.50	40.20	28.76	63.13	74.85	0.30	0.46	0.15	e
92	ZnTe	s8	71.50	40.80	31.10	23.43	51.03	60.93	0.30	0.46	0.16	e
		s9	71.34	40.78	31.15	23.40	50.97	60.87	0.30	0.46	0.16	e
93	$Al_{10}V$	s10	113.70	72.90	62.30	39.87	86.50	103.44	0.30	0.46	0.10	c
		s1	116.00	69.00	62.00	42.03	84.67	108.03	0.29	0.50	0.06	c
94	$Y_2Ti_2O_7$	s48	325.20	111.70	73.20	85.17	182.87	221.17	0.30	0.47	0.17	c
95	InP	s8	102.20	57.60	46.00	34.40	72.47	89.06	0.30	0.47	0.13	e
		s18	101.10	56.10	45.60	34.34	71.10	88.70	0.29	0.48	0.12	e
		s18	102.00	49.30	48.80	38.11	66.87	96.05	0.26	0.57	0.01	c
96	RbBr	s8	31.50	4.80	3.82	6.49	13.70	16.75	0.30	0.47	0.06	e
		s9	31.52	5.00	3.80	6.45	13.84	16.69	0.30	0.47	0.07	e
97	RbCl	s8	36.40	6.30	4.70	7.66	16.33	19.82	0.30	0.47	0.08	e
		s9	36.24	6.12	4.68	7.65	16.16	19.76	0.30	0.47	0.07	e
98	V_3Si	s8	287.00	119.00	81.10	82.25	175.00	213.32	0.30	0.47	0.18	e
99	$Y_3Fe_2(FeO_4)_5$	s9	268.00	110.60	76.60	77.43	163.07	200.55	0.30	0.47	0.17	e
100	NaBeB	s49	106.44	42.40	30.20	30.92	63.75	79.84	0.29	0.48	0.15	c

101	$HfCo_2$	s8	256.00	123.00	90.30	79.89	167.33	206.75	0.29	0.48	0.16	e
102	$ZrCo_2$	s8	233.00	113.00	83.70	73.25	153.00	189.50	0.29	0.48	0.15	e
103	EuF_2	s8	107.00	43.00	29.90	30.72	64.33	79.51	0.29	0.48	0.16	e
104	CaF_2	s8	165.00	47.00	33.90	42.40	86.33	109.28	0.29	0.49	0.12	e
105	SrF_2	s8	124.00	44.00	31.80	34.86	70.67	89.81	0.29	0.49	0.14	e
		s9	123.50	43.05	31.28	34.60	69.87	89.08	0.29	0.50	0.13	e
106	Al_4Cu_9	s10	223.40	80.30	58.30	63.28	128.00	162.99	0.29	0.49	0.13	c
107	YS	s8	249.00	29.00	28.00	50.35	102.33	129.09	0.29	0.49	0.01	e
108	InSb	s9	67.20	36.70	30.20	22.96	46.87	59.18	0.29	0.49	0.11	e
		s18	66.69	36.45	30.20	22.88	46.53	58.95	0.29	0.49	0.11	e
		s18	66.90	36.70	30.70	23.09	46.77	59.46	0.29	0.49	0.10	c
		s8	66.20	35.90	30.20	22.90	46.00	58.89	0.29	0.50	0.10	e
109	$SnNMn_3$	s43	466.73	134.58	100.30	122.90	245.30	315.89	0.29	0.50	0.11	c
		s43	552.69	152.88	112.18	141.67	286.15	364.73	0.29	0.50	0.11	c
110	YMg	s40	51.80	35.50	37.30	20.49	40.93	52.28	0.29	0.50	-0.03	c
		s40	53.30	36.40	39.00	21.36	42.03	54.36	0.28	0.51	-0.05	c
111	InAs	s8	84.40	46.40	39.60	29.49	59.07	75.81	0.29	0.50	0.09	e
		s9	83.29	45.26	39.59	29.50	57.94	75.61	0.28	0.51	0.07	e
		s18	83.29	45.26	39.59	29.50	57.94	75.61	0.28	0.51	0.07	e
		s18	89.00	44.90	41.90	32.38	59.60	82.23	0.27	0.54	0.04	c
112	KI	s8	27.40	4.30	3.70	5.96	12.00	15.30	0.29	0.50	0.04	e
113	ThO_2	s8	367.00	106.00	79.70	97.21	193.00	249.68	0.28	0.50	0.11	e
114	UC	s8	318.00	86.20	65.60	82.55	163.47	211.93	0.28	0.50	0.10	e
		s9	320.00	85.00	64.70	82.35	163.33	211.46	0.28	0.50	0.10	e
115	NbC	s50	620.00	200.00	150.00	171.68	340.00	440.82	0.28	0.50	0.11	e
		s50	640.00	180.00	140.00	170.99	333.33	438.02	0.28	0.51	0.09	c
116	Mg_2Ce	s51	56.88	27.44	21.60	18.52	37.25	47.67	0.29	0.50	0.12	c
		s51	56.57	27.56	21.77	18.50	37.23	47.61	0.29	0.50	0.12	c

117	Rh_3Ti	s52	309.00	174.00	156.00	111.47	219.00	285.66	0.28	0.51	0.06	c
118	TiMoN	s53	573.33	191.23	145.09	162.01	318.60	415.57	0.28	0.51	0.11	c
119	BaO	s8	122.00	45.00	34.40	35.99	70.67	92.29	0.28	0.51	0.11	e
120	USb	s8	195.00	11.00	17.80	36.88	72.33	93.59	0.28	0.51	-0.07	e
121	KBr	s9	34.68	5.80	5.07	7.83	15.43	20.06	0.28	0.51	0.04	e
		s8	34.50	5.50	5.10	7.87	15.17	20.09	0.28	0.52	0.02	e
122	$ZnAl2O_4$	s33	316.00	169.00	148.00	111.75	218.00	286.21	0.28	0.51	0.07	c
		s33	436.00	171.00	139.00	136.36	259.33	348.08	0.28	0.53	0.09	c
123	Rh_3Hf	s52	296.00	158.00	140.00	105.39	204.00	269.60	0.28	0.52	0.07	c
124	KF	s8	65.00	15.00	12.50	16.56	31.67	42.29	0.28	0.52	0.06	e
		s9	64.90	15.20	12.32	16.38	31.77	41.92	0.28	0.52	0.07	e
125	UN	s8	417.00	90.70	75.70	103.52	199.47	264.60	0.28	0.52	0.06	e
126	$FeCr_2O_4$	s9	322.50	143.70	116.70	104.89	203.30	268.49	0.28	0.52	0.10	e
127	KCl	s9	40.69	7.11	6.31	9.46	18.30	24.16	0.28	0.52	0.03	e
		s8	40.50	6.27	6.90	10.02	17.68	25.26	0.26	0.57	-0.02	e
128	$NaBrO_3$	s9	54.50	19.10	15.00	16.03	30.90	40.99	0.28	0.52	0.10	e
129	BSO	s30	129.80	30.20	24.70	32.84	63.40	83.98	0.28	0.52	0.07	e
		s30	116.50	30.10	23.90	30.36	58.90	77.71	0.28	0.52	0.08	c
		s30	150.10	36.50	31.40	39.90	74.37	101.52	0.27	0.54	0.05	c
130	$AlZr_3$	s14	148.65	79.39	70.83	53.15	102.48	135.89	0.28	0.52	0.06	c
		s14	163.80	79.30	86.50	64.88	107.47	161.95	0.25	0.60	-0.04	c
131	Rh_3Zr	s52	300.00	150.00	133.00	105.69	200.00	269.53	0.28	0.53	0.06	c
132	NaI	s8	30.20	9.00	7.36	8.52	16.07	21.72	0.27	0.53	0.08	e
		s9	30.07	9.12	7.33	8.46	16.10	21.60	0.28	0.53	0.08	e
133	$NaClO_3$	s9	49.20	14.20	11.60	13.68	25.87	34.90	0.28	0.53	0.07	e
134	SnTe	s9	112.50	7.50	11.72	22.52	42.50	56.97	0.28	0.53	-0.07	e
135	AlSb	s17	89.00	38.40	41.30	33.93	55.27	84.49	0.25	0.61	-0.03	c
		s8	87.70	43.40	40.80	31.93	58.17	80.95	0.27	0.55	0.03	e

		s18	84.40	43.20	39.50	30.42	56.93	77.43	0.27	0.53	0.05	c
		s9	89.39	44.27	41.55	32.52	59.31	82.46	0.27	0.55	0.03	e
136	$Mg_{24}Y_5$	s40	73.90	22.10	18.00	20.83	39.37	53.12	0.28	0.53	0.08	e
137	CeN	s54	328.80	70.00	60.80	82.70	156.27	210.78	0.28	0.53	0.04	c
		s54	310.20	83.20	72.60	86.89	158.87	220.47	0.27	0.55	0.05	c
138	$NbCRu_3$	s57	566.00	178.00	147.00	164.29	307.33	418.32	0.27	0.53	0.07	c
139	$TaCRu_3$	s57	546.00	187.00	150.00	161.18	306.67	411.45	0.28	0.53	0.09	c
140	TiWN	s53	574.01	154.70	132.14	159.11	294.47	404.45	0.27	0.54	0.06	c
141	$CaSnO_3$	s47	409.97	89.41	79.94	106.03	196.26	269.44	0.27	0.54	0.04	c
142	PbS	s 8	127.00	24.40	23.00	31.92	58.60	80.98	0.27	0.54	0.02	e
143	$PtSb_2$	s 8	260.00	68.00	59.00	71.78	132.00	182.27	0.27	0.54	0.05	e
144	Mg_2La	s40	58.40	24.90	21.80	19.62	36.07	49.82	0.27	0.54	0.06	c
145	GaSb	s18	86.80	44.10	40.70	31.42	58.33	79.88	0.27	0.54	0.04	c
		s8	88.40	40.30	43.40	34.25	56.33	85.41	0.25	0.61	-0.04	e
		s9	88.39	40.33	43.16	34.12	56.35	85.15	0.25	0.61	-0.03	e
		s18	88.34	40.23	43.22	34.16	56.27	85.22	0.25	0.61	-0.04	e
146	TbZn	s46	105.17	33.47	28.16	31.02	57.37	78.85	0.27	0.54	0.07	c
		s46	82.39	30.75	26.37	26.15	47.96	66.38	0.27	0.55	0.07	c
		s46	82.84	30.71	30.71	28.76	48.09	71.94	0.25	0.60	0.00	e
147	CsCl	s8	36.60	9.00	8.07	10.02	18.20	25.39	0.27	0.55	0.04	e
		s9	36.44	8.82	8.04	10.00	18.03	25.32	0.27	0.55	0.03	e
148	NH_4Br	s9	34.14	7.82	7.22	9.20	16.59	23.30	0.27	0.55	0.03	e
149	TaC	s9	505.00	73.00	79.00	119.83	217.00	303.06	0.27	0.55	-0.02	e
		s56	641.00	146.00	156.00	187.84	311.00	469.03	0.25	0.60	-0.02	c
		s56	621.00	155.30	166.80	190.68	310.53	474.84	0.25	0.61	-0.02	c
150	Mg_3As_2	s40	76.20	36.10	32.90	26.97	49.47	68.47	0.27	0.55	0.05	c
151	AlY	s10	81.80	54.35	64.70	35.17	63.50	88.27	0.27	0.55	-0.12	c
152	$MgAl_2O_4$	s30	282.90	155.40	154.80	108.45	197.90	274.79	0.27	0.55	0.00	e

		s9	298.57	153.72	157.58	115.35	202.00	290.51	0.26	0.57	-0.01	e
		s30	273.60	149.60	150.70	105.53	190.93	267.05	0.27	0.55	0.00	c
		s30	256.50	133.20	142.40	101.77	174.30	255.34	0.26	0.58	-0.04	c
153	Zr_3N_4	s57	454.40	165.30	145.90	145.36	261.67	367.94	0.27	0.56	0.05	c
		s57	373.70	140.60	138.60	129.31	218.30	323.97	0.25	0.59	0.01	c
154	$Sr_8Ga_{16}Ge_{30}$	s58	105.50	43.30	39.80	36.06	64.03	91.08	0.26	0.56	0.04	c
155	UCd_{11}	s8	100.70	35.80	32.40	32.42	57.43	81.86	0.26	0.56	0.04	e
156	CsBr	s8	30.70	8.40	7.49	8.79	15.83	22.25	0.27	0.56	0.04	e
		s9	30.63	8.07	7.50	8.84	15.59	22.30	0.26	0.57	0.03	e
157	UAs	s8	250.00	10.00	26.00	50.73	90.00	127.02	0.26	0.56	-0.13	e
158	LiI	s9	28.50	14.00	13.50	10.52	18.83	26.60	0.26	0.56	0.02	e
159	MgCe	s51	47.47	30.42	35.41	20.16	36.10	50.65	0.27	0.56	-0.10	c
		s51	49.20	29.75	35.74	21.30	36.23	53.17	0.26	0.59	-0.11	c
160	Al_2Nd	s8	141.00	47.00	42.80	44.43	78.33	112.10	0.26	0.57	0.04	e
161	Al_3V	s1	178.00	88.00	87.00	66.77	118.00	168.47	0.26	0.57	0.01	c
162	AlN	s20	328.00	139.00	133.00	115.98	202.00	292.04	0.26	0.57	0.02	c
		s20	346.00	146.00	167.00	135.95	212.67	336.16	0.24	0.64	-0.06	c
163	AlP	s20	132.00	63.00	62.00	49.01	86.00	123.52	0.26	0.57	0.01	e
164	Al_7Sr_8	s21	52.16	14.57	13.52	15.43	27.10	38.90	0.26	0.57	0.03	c
		s10	53.60	14.70	14.80	16.51	27.67	41.32	0.25	0.60	-0.00	c
165	AlAs	s19	119.30	57.20	57.20	44.76	77.90	112.67	0.26	0.57	0.00	c
		s17	123.50	53.50	57.50	47.12	76.83	117.35	0.25	0.61	-0.03	c
		s18	119.90	57.50	56.60	44.57	78.30	112.36	0.26	0.57	0.01	e
		s18	116.00	55.00	57.00	44.35	75.33	111.19	0.25	0.59	-0.02	c
		s18	119.00	55.00	57.30	45.36	76.33	113.55	0.25	0.59	-0.02	c
166	Ir_3Zr	s59	398.00	198.00	196.00	149.61	264.67	377.52	0.26	0.57	0.01	c
		s28	441.46	202.87	220.51	172.33	282.40	429.48	0.25	0.61	-0.04	c

167	Fe_2VGa	s60	363.10	161.00	152.30	129.21	228.37	326.10	0.26	0.57	0.03	c
168	Hf_3N_4	s57	493.20	167.30	152.40	156.54	275.93	394.93	0.26	0.57	0.04	c
		s57	399.30	142.50	145.10	138.17	228.10	344.88	0.25	0.61	-0.01	c
169	$CeSn_3$	s8	80.60	42.10	42.80	31.06	54.93	78.34	0.26	0.57	-0.01	e
170	NaBi	s8	40.00	10.60	9.96	11.65	20.40	29.35	0.26	0.57	0.02	e
171	PbTe	s9	107.95	7.64	13.43	23.56	41.08	59.03	0.26	0.57	-0.10	e
172	Fe_3O_4	s9	273.00	106.00	97.10	91.41	161.67	230.74	0.26	0.57	0.04	e
173	r-Sn_3N_4	s30	282.10	139.50	140.40	106.97	187.03	269.42	0.26	0.57	0.00	c
		s30	246.10	116.90	128.00	97.28	159.97	242.55	0.25	0.61	-0.05	c
174	YSb	s61	149.50	19.30	24.00	36.27	62.70	91.07	0.26	0.58	-0.05	c
175	YZn	s8	94.40	46.00	47.30	36.15	62.13	90.79	0.26	0.58	-0.01	e
176	CsI	s8	24.50	6.60	6.31	7.26	12.57	18.26	0.26	0.58	0.02	e
		s9	24.46	6.61	6.29	7.24	12.56	18.21	0.26	0.58	0.02	e
177	LiBr	s3	39.40	18.90	19.10	14.88	25.73	37.41	0.26	0.58	-0.01	e
		s9	39.40	18.80	19.10	14.91	25.67	37.46	0.26	0.58	-0.01	e
178	YAG	s30	356.70	122.60	114.30	115.39	200.63	290.49	0.26	0.58	0.03	c
		s30	333.00	113.00	115.00	112.97	186.33	281.94	0.25	0.61	-0.01	e
179	Rh_3Nb	s54	395.00	172.00	175.00	146.07	246.33	365.85	0.25	0.59	-0.01	c
180	HoBi	s62	133.05	16.66	21.73	32.66	55.46	81.75	0.25	0.59	-0.06	c
181	$SrHfO_3$	s63	397.58	79.41	85.42	109.86	185.47	275.16	0.25	0.59	-0.02	c
182	NaCl	s8	49.10	12.80	12.80	14.73	24.90	36.90	0.25	0.59	0.00	e
		s9	49.47	12.88	12.87	14.82	25.08	37.14	0.25	0.59	0.00	e
183	NaBr	s9	39.70	10.01	9.98	11.71	19.91	29.36	0.25	0.59	0.00	e
184	TiN	s30	625.00	165.00	163.00	187.15	318.33	469.44	0.25	0.59	0.00	e
		s53	713.00	133.00	166.00	207.92	326.33	514.40	0.24	0.64	-0.06	c
		s30	680.00	130.00	171.00	207.04	313.33	508.95	0.23	0.66	-0.08	c
		s64	596.10	124.90	154.70	183.20	281.97	451.72	0.23	0.65	-0.07	c
		s30	688.00	124.00	171.00	209.18	312.00	512.84	0.23	0.67	-0.09	c

		s53	671.00	106.40	166.20	205.78	294.60	500.67	0.22	0.70	-0.12	c
185	InN	s20	217.00	101.00	104.00	82.28	139.67	206.27	0.25	0.59	-0.01	c
186	$VCRu_3$	s57	615.00	143.00	146.00	177.14	300.33	444.06	0.25	0.59	-0.01	c
187	Ir_3Hf	s28	442.88	206.53	221.72	172.24	285.31	430.03	0.25	0.60	-0.04	c
188	ErSb	s8	150.00	20.00	25.80	37.74	63.33	94.33	0.25	0.60	-0.06	e
189	PrSb	s8	122.00	14.00	19.80	30.00	50.00	74.86	0.25	0.60	-0.08	e
190	NH_4Cl	s9	38.14	8.66	9.03	11.00	18.49	27.53	0.25	0.60	-0.01	e
191	Mg_2Pb	s40	55.20	23.30	24.30	20.53	33.93	51.24	0.25	0.60	-0.02	c
		s8	71.70	22.10	30.90	28.30	38.63	68.23	0.21	0.73	-0.13	e
		s40	71.70	22.10	30.90	28.30	38.63	68.23	0.21	0.73	-0.13	c
192	GaAs	s18	120.00	55.20	57.80	45.82	76.80	114.63	0.25	0.60	-0.02	c
		s8	118.00	53.50	59.40	46.49	75.00	115.55	0.24	0.62	-0.05	e
		s9	118.77	53.72	59.44	46.67	75.40	116.03	0.24	0.62	-0.05	e
		s18	119.00	53.80	59.50	46.74	75.53	116.21	0.24	0.62	-0.05	e
		s18	123.00	53.00	62.00	49.29	76.33	121.65	0.23	0.65	-0.07	c
193	Rh_3Ta	s52	408.00	170.00	180.00	152.49	249.33	379.97	0.25	0.61	-0.03	c
194	HoSb	s62	150.23	17.48	25.25	37.63	61.73	93.68	0.25	0.61	-0.08	c
195	$SnNCa_3$	s65	104.00	44.00	47.00	39.26	64.00	97.77	0.25	0.61	-0.03	c
		s65	90.00	39.00	44.00	35.35	56.00	87.60	0.24	0.63	-0.06	c
196	$PbNCa_3$	s65	75.00	38.00	43.00	30.65	50.33	76.37	0.25	0.61	-0.07	c
		s65	98.00	43.00	47.00	37.91	61.33	94.28	0.24	0.62	-0.04	c
197	$AsNBa_3$	s66	90.41	23.33	24.76	27.96	45.69	69.68	0.25	0.61	-0.02	c
		s67	68.71	15.38	19.36	22.01	33.16	54.07	0.23	0.66	-0.07	c
198	LiCl	s9	49.27	23.10	24.95	19.26	31.82	48.06	0.25	0.61	-0.04	e
		s8	49.10	22.00	24.80	19.46	31.03	48.27	0.24	0.63	-0.06	e
199	$Al_{12}Mo$	This work	167.20	56.30	59.50	57.85	93.27	143.81	0.24	0.62	-0.02	c
		s112	167.07	54.64	61.80	59.50	92.12	146.88	0.23	0.65	-0.04	c

200	$Al_{12}W$	This work	171.80	58.10	60.80	59.19	96.00	147.29	0.24	0.62	-0.02	c
		s112	168.28	56.00	60.66	58.81	93.43	145.83	0.24	0.63	-0.03	c
201	GaP	s18	140.00	61.60	68.50	54.76	87.73	135.95	0.24	0.62	-0.05	c
		s8	141.00	62.40	71.20	56.09	88.60	138.92	0.24	0.63	-0.06	e
		s9	141.20	62.53	70.47	55.77	88.75	138.30	0.24	0.63	-0.06	e
		s18	140.50	62.03	70.33	55.65	88.19	137.90	0.24	0.63	-0.06	e
		s18	147.00	61.00	79.00	61.89	89.67	150.90	0.22	0.69	-0.12	c
202	$ZnIn_2O_4$	s33	331.95	107.66	112.93	112.62	182.42	280.19	0.24	0.62	-0.02	c
203	$CeIn_3$-200k	s8	92.90	33.90	37.30	33.96	53.57	84.10	0.24	0.63	-0.04	e
204	$Mg_{23}Ba_6$	s40	41.50	15.90	17.20	15.28	24.43	37.93	0.24	0.63	-0.03	c
205	$SrTiO_3$	s30	397.20	100.80	114.10	126.70	199.60	313.72	0.24	0.63	-0.04	c
		s30	324.70	89.80	106.30	110.63	168.10	272.18	0.23	0.66	-0.06	c
		s9	334.00	104.00	126.00	121.48	180.67	297.71	0.23	0.67	-0.07	e
		s30	335.00	105.00	127.00	122.06	181.67	299.17	0.23	0.67	-0.07	e
206	HfN	s30	679.00	119.00	150.00	193.11	305.67	478.40	0.24	0.63	-0.06	e
207	Al_2Pr	s8	138.00	41.80	45.20	46.34	73.87	114.97	0.24	0.63	-0.03	e
208	Al_3Zr	s22	175.00	62.60	69.60	63.89	100.07	158.04	0.24	0.64	-0.04	c
209	NiO	s8	225.00	95.00	110.00	89.07	138.33	219.96	0.23	0.64	-0.07	e
210	NaF	s8	97.00	24.20	28.10	31.17	48.47	77.00	0.24	0.64	-0.05	e
		s9	97.00	23.80	28.22	31.32	48.20	77.23	0.23	0.65	-0.06	e
211	r-Mg_2SiO_4	s30	327.00	112.00	126.00	118.24	183.67	292.05	0.23	0.64	-0.05	c
		s30	338.30	111.80	130.00	123.02	187.30	302.77	0.23	0.66	-0.06	c
		s30	299.60	103.30	128.20	115.20	168.73	281.52	0.22	0.68	-0.09	e
212	GaN	s20	305.00	128.00	147.00	119.93	187.00	296.38	0.24	0.64	-0.06	c
		s20	296.00	154.00	206.00	134.50	201.33	329.19	0.23	0.67	-0.16	c
213	$Al_{12}Cr$	This work	153.80	53.90	61.00	56.31	87.20	139.01	0.23	0.65	-0.05	c

214	Al_3Ti	s1	192.00	65.00	74.00	69.60	107.33	171.70	0.23	0.65	-0.05	c
		s22	185.20	62.50	73.20	68.21	103.40	167.73	0.23	0.66	-0.06	c
215	AlCo	s10	301.10	119.10	140.70	118.15	179.77	290.72	0.23	0.66	-0.07	c
216	$BaSnO_3$	s68	358.50	93.50	110.30	118.70	181.83	292.46	0.23	0.65	-0.06	c
		s68	285.20	68.50	84.30	93.22	140.73	229.07	0.23	0.66	-0.07	c
217	TiCrN	s53	649.40	123.90	160.22	195.54	299.07	481.59	0.23	0.65	-0.08	c
218	$GaMgF_3$	s69	127.70	37.72	43.65	44.18	67.71	108.87	0.23	0.65	-0.05	c
		s69	138.00	43.60	49.83	48.76	75.07	120.25	0.23	0.65	-0.05	e
		s69	138.50	44.10	50.01	48.87	75.57	120.60	0.23	0.65	-0.05	e
		s69	177.00	48.70	58.70	60.82	91.47	149.36	0.23	0.66	-0.07	c
		s69	132.00	39.60	48.50	47.57	70.40	116.47	0.22	0.68	-0.08	e
		s69	137.00	39.50	54.60	52.18	72.00	126.08	0.21	0.72	-0.12	c
		s69	119.26	38.26	63.23	52.89	65.26	124.91	0.18	0.81	-0.20	c
219	$TlNCa_3$	s70	105.70	35.40	40.50	38.27	58.83	94.35	0.23	0.65	-0.05	c
		s70	82.20	30.30	37.10	32.15	47.60	78.72	0.22	0.68	-0.09	c
220	$GeNCa_3$	s65	119.00	39.00	45.00	42.93	65.67	105.74	0.23	0.65	-0.06	c
		s65	102.00	36.00	43.00	38.68	58.00	94.93	0.23	0.67	-0.07	c
221	Rh_3V	s52	378.00	148.00	176.00	148.40	224.67	364.83	0.23	0.66	-0.08	c
222	Ir_3Ta	s28	655.32	259.01	311.31	259.74	391.11	637.93	0.23	0.66	-0.08	c
223	Ir_3Nb	s28	631.94	252.94	303.39	251.21	379.27	617.27	0.23	0.66	-0.08	c
224	VC	s71	578.20	147.20	176.30	191.06	290.87	470.22	0.23	0.66	-0.06	c
225	Al_2Ce	s23	139.00	33.70	41.90	45.91	68.80	112.67	0.23	0.67	-0.07	c
		s8	146.60	29.80	43.70	49.09	68.73	118.94	0.21	0.71	-0.12	e
226	Al_2La	s23	134.50	32.80	41.00	44.69	66.70	109.60	0.23	0.67	-0.07	c
		s8	145.40	33.00	43.00	47.87	70.47	117.09	0.22	0.68	-0.09	e
227	TiVN	s53	574.80	123.90	159.18	183.04	274.20	449.16	0.23	0.67	-0.08	c
228	TiAlN	s53	503.87	143.05	174.01	176.54	263.32	432.89	0.23	0.67	-0.07	c
229	YAs	s61	177.80	24.40	38.00	50.54	75.53	123.92	0.23	0.67	-0.11	c

230	SrO	s8	170.00	46.00	55.60	58.08	87.33	142.62	0.23	0.67	-0.07	e
		s9	160.10	43.50	59.00	58.72	82.37	142.33	0.21	0.71	-0.11	e
231	Mg_2Sn	s40	69.80	25.90	31.10	27.05	40.53	66.38	0.23	0.67	-0.08	c
		s72	81.10	20.16	34.85	33.03	40.47	77.89	0.18	0.82	-0.19	c
		s72	82.40	20.80	36.60	34.16	41.33	80.34	0.18	0.83	-0.20	c
		s8	82.40	20.80	36.60	34.16	41.33	80.34	0.18	0.83	-0.20	e
		s40	82.40	20.80	36.60	34.16	41.33	80.34	0.18	0.83	-0.20	c
232	TiNbN	s53	591.76	102.85	146.58	180.13	265.82	440.76	0.22	0.68	-0.10	c
233	Ir_3Ti	s28	584.52	226.65	281.48	234.73	345.94	574.24	0.22	0.68	-0.10	c
234	Mg_3Ce	s51	58.34	27.71	36.42	25.73	37.92	62.88	0.22	0.68	-0.14	c
235	TiC	s73	610.00	124.00	173.00	198.27	286.00	483.14	0.22	0.69	-0.10	c
		s73	527.00	112.00	159.00	176.90	250.33	429.52	0.21	0.71	-0.11	c
		s9	500.00	113.00	175.00	182.18	242.00	436.90	0.20	0.75	-0.14	e
		s73	513.00	106.00	178.00	187.80	241.67	447.48	0.19	0.78	-0.16	e
		s73	470.00	97.00	167.00	174.54	221.33	414.64	0.19	0.79	-0.17	c
236	YP	s61	199.40	28.70	46.00	59.07	85.60	144.03	0.22	0.69	-0.12	c
237	$Ba_8Ga_{16}Ge_{30}$	s58	116.30	39.70	50.40	45.15	65.23	110.06	0.22	0.69	-0.10	c
238	CoSi	s8	328.00	95.00	120.00	118.59	172.67	289.49	0.22	0.69	-0.09	e
239	Ir_3V	s28	560.69	213.39	281.33	231.84	329.16	563.20	0.21	0.70	-0.12	c
240	$SbNBa_3$	s67	77.12	15.90	22.27	25.30	36.31	61.59	0.22	0.70	-0.10	c
		s66	98.18	18.80	27.86	32.11	45.26	77.91	0.21	0.71	-0.12	c
241	ScN	s74	396.75	130.92	169.58	153.82	219.53	374.09	0.22	0.70	-0.10	c
242	CeB_6	s8	508.00	19.00	79.00	126.77	182.00	307.64	0.22	0.70	-0.20	e
		s8	473.00	16.00	81.00	124.60	168.33	299.09	0.20	0.74	-0.22	e
243	CaO	s8	224.00	60.00	80.60	81.16	114.67	197.00	0.21	0.71	-0.10	e
		s30	224.00	60.00	80.60	81.16	114.67	197.00	0.21	0.71	-0.10	e
		s30	207.80	49.90	79.50	79.28	102.53	189.10	0.19	0.77	-0.16	c

		s30	240.50	48.20	93.20	94.37	112.30	221.16	0.17	0.84	-0.20	c
244	Mg_3La	s40	59.20	26.20	36.00	26.32	37.20	63.85	0.21	0.71	-0.15	c
245	$InNCo_3$	s75	317.54	94.98	126.76	120.32	169.17	291.79	0.21	0.71	-0.11	c
		s75	302.00	74.00	141.00	129.50	150.00	301.68	0.16	0.86	-0.22	c
246	Al_2U	s8	170.40	39.20	54.80	58.89	82.93	142.86	0.21	0.71	-0.11	e
247	Al_2Lu	s23	174.30	38.10	54.90	59.85	83.50	144.92	0.21	0.72	-0.12	c
248	Al_3La	s24	123.10	32.40	44.90	45.08	62.63	109.07	0.21	0.72	-0.11	c
249	TiZrN	s53	593.89	85.07	147.15	183.53	254.68	443.85	0.21	0.72	-0.14	c
250	Fe_2VAl	s60	415.70	125.30	170.70	160.00	222.10	387.05	0.21	0.72	-0.12	c
251	BeO	s8	381.00	147.00	200.00	161.30	225.00	390.49	0.21	0.72	-0.14	e
252	LiF	s8	112.00	46.00	63.50	48.83	68.00	118.15	0.21	0.72	-0.15	e
253	TiMoC	s73	596.00	113.00	176.00	199.81	274.00	482.20	0.21	0.73	-0.13	c
254	TiTaC	s73	574.00	119.00	180.00	197.70	270.67	476.97	0.21	0.73	-0.13	c
255	TiNbC	s73	560.00	116.00	176.00	193.15	264.00	465.84	0.21	0.73	-0.13	c
256	TiZrC	s73	508.00	103.00	157.00	173.85	238.00	419.43	0.21	0.73	-0.13	c
257	Al_2Y	s23	168.60	34.80	54.40	59.10	79.40	142.05	0.20	0.74	-0.14	c
		s8	170.80	34.00	56.20	60.80	79.60	145.38	0.20	0.76	-0.15	e
		s10	172.70	33.80	56.30	61.24	80.10	146.40	0.20	0.76	-0.15	c
258	TiVC	s73	549.00	115.00	177.00	192.04	259.67	462.19	0.20	0.74	-0.13	c
259	$InCCo_3$	s75	378.00	89.00	133.00	137.49	185.33	330.69	0.20	0.74	-0.13	c
260	TiHfC	s73	517.00	103.00	165.00	180.69	241.00	433.68	0.20	0.75	-0.14	c
261	Be_2B	s49	243.72	110.46	168.42	116.12	154.88	278.27	0.20	0.75	-0.21	c
262	Al_3Sc	s25	189.10	43.20	66.10	68.76	91.83	165.08	0.20	0.75	-0.14	c
263	AlSr	s21	62.34	12.98	21.12	22.48	29.43	53.75	0.20	0.76	-0.15	c
264	ZrC	s76	470.00	100.00	160.00	169.57	223.33	405.97	0.20	0.76	-0.15	e
		s76	472.00	99.00	159.00	169.48	223.33	405.80	0.20	0.76	-0.15	e
		s9	472.00	98.70	159.30	169.73	223.13	406.19	0.20	0.76	-0.15	e
		s76	499.00	93.00	170.00	182.51	228.33	432.34	0.18	0.80	-0.18	c

		s76	504.00	90.00	173.00	185.88	228.00	438.48	0.18	0.82	-0.19	c
		s8	441.00	60.00	151.00	165.73	187.00	383.80	0.16	0.89	-0.24	e
265	YN	s61	309.90	80.70	124.00	120.15	157.10	287.23	0.20	0.76	-0.15	e
266	BP	s18	308.00	107.00	162.00	133.77	174.00	319.41	0.19	0.77	-0.17	c
		s8	315.00	100.00	160.00	136.43	171.67	323.55	0.19	0.79	-0.19	e
		s18	315.00	100.00	160.00	136.43	171.67	323.55	0.19	0.79	-0.19	e
267	Al_2Sc	s23	186.80	40.90	65.70	68.51	89.53	163.76	0.20	0.77	-0.15	c
268	Al_2Gd	s8	160.50	36.80	59.50	60.43	78.03	144.09	0.19	0.77	-0.16	e
269	Al_3Ce	s24	134.90	30.10	49.10	50.39	65.03	120.15	0.19	0.77	-0.16	c
270	Al_3Lu	s24	159.90	38.40	60.40	60.54	78.90	144.63	0.19	0.77	-0.15	c
271	Al_2Ca	s8	97.00	22.40	36.60	36.88	47.27	87.80	0.19	0.78	-0.16	e
272	TiCrC	s73	443.00	98.00	161.00	165.51	213.00	394.37	0.19	0.78	-0.16	c
273	$AsNSr_3$	s77	134.00	18.00	37.00	44.33	56.67	105.48	0.19	0.78	-0.18	c
274	Sc_3AlN	s74	234.32	54.21	87.76	88.67	114.25	211.34	0.19	0.78	-0.16	c
275	$AsNSr_3$	s77	110.00	16.90	35.80	39.77	47.93	93.46	0.18	0.83	-0.20	c
276	Al_3Sc	s24	181.60	41.60	69.30	69.58	88.27	165.30	0.19	0.79	-0.17	c
277	Sc_3InN	s74	238.57	54.28	90.76	91.31	115.71	216.88	0.19	0.79	-0.17	c
278	$Al_{12}Re$	This work	197.10	49.60	83.40	79.40	98.77	187.85	0.18	0.80	-0.18	c
		s112	184.55	57.19	88.34	77.49	99.64	184.61	0.19	0.78	-0.17	c
279	Al_2Eu	s8	104.70	23.90	40.70	40.58	50.83	96.15	0.18	0.80	-0.17	e
280	$SnBSc_3$	s78	224.20	32.00	65.80	76.61	96.07	181.55	0.19	0.80	-0.19	c
		s78	205.70	28.60	63.50	72.56	87.63	170.59	0.18	0.83	-0.20	c
281	$PbBSc_3$	s78	211.00	30.80	64.10	73.48	90.87	173.63	0.18	0.81	-0.19	c
		s78	197.10	28.00	61.50	69.87	84.37	164.27	0.18	0.83	-0.20	c
282	$AlCY_3$	s38	179.30	33.40	62.90	66.74	82.03	157.51	0.18	0.81	-0.19	c
283	MgO	s8	294.00	93.00	155.00	130.29	160.00	307.39	0.18	0.81	-0.20	e
		s9	297.08	95.36	156.13	131.04	162.60	309.85	0.18	0.81	-0.20	e

		s30	291.00	92.00	156.00	130.26	158.33	306.65	0.18	0.82	-0.21	c
		s30	276.00	86.00	149.00	124.40	149.33	292.06	0.17	0.83	-0.22	c
284	SiC	s8	352.00	140.00	233.00	169.86	210.67	401.27	0.18	0.81	-0.23	e
285	$Al_{12}Mn$	This work	190.10	41.80	75.50	74.96	91.23	176.53	0.18	0.82	-0.19	c
		s10	181.70	46.50	73.20	70.91	91.57	169.08	0.19	0.77	-0.16	c
286	Al_3Y	s24	158.60	33.20	61.00	61.67	75.00	145.22	0.18	0.82	-0.19	c
287	$SiGe_2N_4$	s79	494.00	172.00	288.00	228.06	279.33	537.67	0.18	0.82	-0.22	c
288	$PNCa_3$	s80	151.00	23.00	48.00	53.87	65.67	126.90	0.18	0.82	-0.20	c
		s80	176.00	22.00	51.00	60.18	73.33	141.76	0.18	0.82	-0.20	c
289	$AsNCa_3$	s80	168.00	24.00	52.00	59.25	72.00	139.48	0.18	0.82	-0.20	c
		s80	146.00	23.00	50.00	54.32	64.00	127.02	0.17	0.85	-0.21	c
290	$SbNSr_3$	s77	112.00	21.00	40.00	42.12	51.33	99.21	0.18	0.82	-0.19	c
		s77	103.00	20.00	38.00	39.36	47.67	92.60	0.18	0.83	-0.19	c
		s80	134.00	28.00	53.00	53.00	63.33	124.32	0.17	0.84	-0.20	c
		s80	149.00	28.00	54.00	56.51	68.33	132.90	0.18	0.83	-0.20	c
291	Sc_3GaN	s74	268.56	47.51	92.16	99.11	121.19	233.65	0.18	0.82	-0.19	c
292	$BiNSr_3$	s77	115.00	20.00	39.80	42.72	51.67	100.47	0.18	0.83	-0.20	c
		s77	96.00	19.00	37.00	37.59	44.67	88.07	0.17	0.84	-0.20	c
293	Mg_2Si	s72	126.00	26.00	48.50	49.09	59.33	115.44	0.18	0.83	-0.19	c
294	$Al_{12}Tc$	This work	194.70	44.60	81.80	79.03	94.63	185.46	0.17	0.84	-0.20	c
295	$BiNCa_3$	s80	140.00	26.00	53.00	54.57	64.00	127.47	0.17	0.85	-0.21	c
		s80	124.00	25.00	50.00	49.80	58.00	116.15	0.17	0.86	-0.22	c
296	MgBeB	s49	207.28	54.66	99.20	89.31	105.53	208.97	0.17	0.85	-0.21	c
298	Mg_2Si	s72	118.82	22.27	44.96	46.26	54.45	108.15	0.17	0.85	-0.21	c
		s40	116.70	23.10	45.30	45.89	54.30	107.42	0.17	0.85	-0.21	c
		s8	121.00	22.00	46.40	47.62	55.00	110.86	0.16	0.87	-0.22	e

		s40	121.00	22.00	46.40	47.62	55.00	110.86	0.16	0.87	-0.22	c
299	La_3S4	s8	116.00	31.60	57.20	50.64	59.73	118.45	0.17	0.85	-0.22	e
300	Li_2O	s8	202.00	21.50	58.70	69.78	81.67	162.92	0.17	0.85	-0.23	c
301	Mg_2Ge	s40	107.30	21.10	41.80	42.32	49.83	98.94	0.17	0.85	-0.21	c
		s40	117.90	23.00	46.50	46.88	54.63	109.36	0.17	0.86	-0.21	c
302	r-Si_3N_4	s30	504.00	177.00	317.00	243.03	286.00	567.89	0.17	0.85	-0.25	c
		s30	529.00	169.00	334.00	260.62	289.00	600.94	0.15	0.90	-0.27	c
303	AlBeB	s49	321.22	62.29	127.73	128.42	148.60	299.10	0.16	0.86	-0.22	c
304	CaF_2	s9	164.20	43.98	84.06	73.49	84.05	170.71	0.16	0.87	-0.23	e
305	Al_2Tb-200k	s8	144.00	33.00	68.00	62.69	70.00	144.83	0.16	0.90	-0.24	e
306	MnSi	s8	293.00	54.00	125.00	122.77	133.67	281.98	0.15	0.92	-0.25	e
307	c-BN	s30	825.00	193.00	475.00	403.41	403.67	907.75	0.13	1.00	-0.31	c
		s30	820.00	190.00	480.00	405.43	400.00	909.05	0.12	1.01	-0.32	e
		s30	783.00	172.00	444.00	382.22	375.67	856.22	0.12	1.02	-0.32	c

Table s3 The theoretical and available experimental elastic constants C_{ij} (GPa), shear G (GPa), bulk B (GPa), Young E (GPa) moduli and Poisson ratio v, Pugh modulus ratio G/B, Cauchy Pressure C_{12}-C_{44} (CP), (C_{12}-C_{44})/E for a series of pure element solid phases with cubic lattices. In the last column, "e" and "c" denotes elastic constants from direct experimental measured and theoretical calculations, respectively.

No.	Compounds	Refs	C_{11}	C_{12}	C_{44}	G	B	E	v	G/B	CP/E	
1	Au	s8	191.00	162.00	42.20	27.52	171.67	78.31	0.42	0.16	1.53	e
		s9	192.44	162.98	42.00	27.61	172.80	78.59	0.42	0.16	1.54	e
2	Pb	s9	49.66	42.31	14.98	8.59	44.76	24.15	0.41	0.19	1.13	e
		s8	48.80	41.40	14.80	8.54	43.87	24.01	0.41	0.19	1.11	e
3	Nb	s9	246.50	134.50	28.73	37.66	171.83	105.27	0.40	0.22	1.00	e
		s8	245.00	132.00	28.40	37.55	169.67	104.88	0.40	0.22	0.99	e
4	Pt	s26	322.40	237.10	74.20	59.42	265.53	165.87	0.40	0.22	0.98	c
		s9	346.70	250.70	76.50	63.46	282.70	177.12	0.40	0.22	0.98	e
		s8	347.00	251.00	76.50	63.46	283.00	177.13	0.40	0.22	0.99	e
5	Al_{12}	This work	99.60	50.70	10.70	15.00	67.00	41.86	0.40	0.22	0.96	c
6	Pd	s26	220.20	174.20	68.73	44.36	189.53	123.30	0.39	0.23	0.86	c
		s9	227.10	176.04	71.73	47.43	193.06	131.38	0.39	0.25	0.79	e
		s8	221.00	171.00	70.80	46.67	187.67	129.15	0.39	0.25	0.78	e
7	Ag	s8	123.99	93.67	46.12	29.56	103.78	80.86	0.37	0.28	0.59	e
		s8	122.00	92.00	45.50	29.20	102.00	79.83	0.37	0.29	0.58	e

		s26	115.80	89.00	40.70	26.10	97.93	71.81	0.38	0.27	0.67	c
		s26	124.00	93.40	46.10	29.66	103.60	81.10	0.37	0.29	0.58	c
8	V	s9	228.70	119.00	43.20	47.54	155.57	129.42	0.36	0.31	0.59	e
		s8	230.00	120.00	43.10	47.52	156.67	129.47	0.36	0.30	0.59	e
9	Cr	s9	339.80	58.60	99.00	113.96	152.33	273.64	0.20	0.75	-0.15	e
10	Li	s9	13.50	11.44	8.78	3.93	12.13	10.47	0.36	0.32	0.25	e
		s8	13.40	11.30	9.60	4.22	12.00	11.09	0.35	0.35	0.15	e
11	K	s9	3.70	3.14	1.88	0.91	3.33	2.47	0.38	0.27	0.51	e
		s8	3.71	3.15	1.88	0.91	3.34	2.47	0.38	0.27	0.51	e
12	Na	s9	7.39	6.22	4.19	1.98	6.61	5.33	0.37	0.30	0.38	e
		s8	7.59	6.33	4.30	2.06	6.75	5.55	0.36	0.31	0.37	e
13	Rb	s9	2.96	2.50	1.71	0.80	2.65	2.15	0.37	0.30	0.37	e
		s8	2.96	2.44	1.60	0.79	2.61	2.14	0.36	0.30	0.39	e
14	Cs-78k	s8	2.47	2.06	1.48	0.70	2.20	1.87	0.36	0.32	0.31	e
15	Al	s1	111.00	57.00	28.00	27.60	75.00	73.74	0.34	0.37	0.39	c
		s2	114.30	61.90	31.60	29.32	79.37	78.31	0.34	0.37	0.39	e
		s8	108.00	62.00	28.30	26.05	77.33	70.25	0.35	0.34	0.48	e
		s9	106.80	60.40	28.30	26.14	75.87	70.33	0.35	0.34	0.46	e
16	Cu	s26	176.50	129.80	82.30	49.83	145.37	133.78	0.35	0.34	0.36	c
		s26	176.40	129.20	75.20	47.34	144.93	127.81	0.35	0.33	0.42	c
		s9	168.30	122.10	75.70	47.14	137.50	126.61	0.35	0.34	0.37	e
		s8	169.00	122.00	75.30	47.30	137.67	127.04	0.35	0.34	0.37	e
17	Fe	s9	226.00	140.00	116.00	77.94	168.67	202.32	0.30	0.46	0.12	e
		s8	230.00	135.00	117.00	81.50	166.67	210.01	0.29	0.49	0.09	e
18	Mo	s9	463.70	157.80	109.20	125.00	259.77	323.17	0.29	0.48	0.15	e
		s8	465.00	163.00	109.00	124.22	263.67	322.08	0.30	0.47	0.17	e
19	W	s9	522.40	204.40	160.80	160.08	310.40	409.79	0.28	0.52	0.11	e
		s8	523.00	203.00	160.00	160.00	309.67	409.48	0.28	0.52	0.11	e

20	Ni	s26	252.70	167.70	116.30	77.71	196.03	205.62	0.33	0.40	0.25	c
		s26	243.60	149.40	119.60	82.31	180.80	214.15	0.30	0.46	0.14	c
		s27	250.00	150.00	131.00	89.05	183.33	229.60	0.29	0.49	0.08	c
		s27	274.00	160.00	130.00	93.38	198.00	241.91	0.30	0.47	0.12	c
		s27	271.00	155.00	127.00	92.73	193.67	239.74	0.29	0.48	0.12	c
		s9	248.10	154.90	124.20	83.85	185.97	218.38	0.30	0.45	0.14	e
		s8	247.00	153.00	122.00	83.23	184.33	216.76	0.30	0.45	0.14	e
21	Ir	s28	634.87	254.51	269.06	234.14	381.30	583.06	0.25	0.61	-0.02	c
		s28	596.00	252.00	270.00	225.34	366.67	561.04	0.24	0.61	-0.03	e
		s28	621.00	256.00	260.00	225.62	377.67	564.45	0.25	0.60	-0.01	c
		s9	580.00	242.00	256.00	216.74	354.67	540.16	0.25	0.61	-0.03	e
		s8	600.00	260.00	270.00	224.29	373.33	560.54	0.25	0.60	-0.02	e
		s26	582.60	229.20	258.20	221.79	347.00	548.49	0.24	0.64	-0.05	c
22	Si	s27	166.00	64.00	79.00	66.29	98.00	162.26	0.22	0.68	-0.09	e
		s27	152.00	56.00	99.00	74.04	88.00	173.35	0.17	0.84	-0.25	c
		s9	165.78	63.94	79.62	66.56	97.89	162.76	0.22	0.68	-0.10	c
		s9	165.00	63.00	79.10	66.34	97.00	162.05	0.22	0.68	-0.10	c
		s29	159.00	67.30	80.00	63.99	97.87	157.58	0.23	0.65	-0.08	c
		s29	151.00	60.60	79.20	63.24	90.73	153.92	0.22	0.70	-0.12	c
23	Ge	s9	128.35	48.23	66.66	54.35	74.94	131.28	0.21	0.73	-0.14	e
		s8	129.00	48.00	67.10	54.80	75.00	132.18	0.21	0.73	-0.14	e
		s29	127.00	44.80	63.10	53.14	72.20	128.00	0.20	0.74	-0.14	c
		s29	103.00	39.00	54.00	43.78	60.33	105.73	0.21	0.73	-0.14	c
24	Diamond	s30	1079.00	124.00	578.00	535.46	442.33	1144.54	0.07	1.21	-0.40	e
		s29	1107.20	144.70	598.10	548.26	465.53	1181.10	0.08	1.18	-0.38	c
		s29	1055.00	120.40	559.00	520.32	431.93	1113.74	0.07	1.21	-0.39	c
		s8	1077.00	124.70	577.00	534.30	442.13	1142.62	0.07	1.21	-0.40	e

Table s4 The theoretical and available experimental shear *G* (GPa), bulk *B* (GPa) moduli, Pugh's modulus ratio *G/B* with the theoretical Vickers hardness value by this work compared with available experimental values of selected compounds, the "refs" column denotes the references of the elastic moduli, and in the last column, "e" and "c" denotes elastic moluli from direct experimental measured and theoretical calculations, respectively. The references for the experimental Vickers hardness values are added right behind it.

No.	Compounds	refs	G	B	G/B	Hv-cal	Hv-exp	
1	diamond	s30	535.46	442.33	1.21	95.7	96[s81]	e
		s29	548.26	465.53	1.18	93.9	60-120[s82]	c
		s29	520.32	431.93	1.20	93.52	115[s83]	c
		s8	534.30	442.13	1.21	95.4	95±5[s84]	e
2	BC_2N	S85	446.00	403.00	1.11	76.9	62,75[s83,s85]	c
		S86	445.00	408.00	1.09	75.4	76±4[s87]	e
3	BC_5	s88	393.66	376.67	1.05	66.4	71[s88]	c
4	c-BN	s30	403.41	403.67	1.00	63.8	47[s89]	c
		s30	405.43	400.00	1.01	65.2	55[s87]	e
		s30	382.22	375.67	1.02	63.1	62[s87]<br>66[s90]<br>63±5[s86]	c
5	r-B_{28}	s91	236.00	224.00	1.05	49.0	50,58±5[s91-s93]	c
6	ReB2	s94	273.00	382.00	0.72	32.9	48±5[s95]	e
		s98	283.00	350.00	0.81	39.4	37.2-40.5[s99]	c
		s101	302.00	371.00	0.81	41.4	28[s99]	c
		s97	289.00	365.00	0.79	38.9	39.3[s96]	c
		s97	276.00	317.00	0.87	42.6	30.1[s100]	e
		s97	295.00	369.00	0.80	39.9	37[s101]<br>30.8-35.8[s102]	c
7	WC	s8	301.80	438.90	0.69	33.4	30[s81]	e
		s81	282.00	439.00	0.64	29.3		e
8	SiC	s8	169.86	210.67	0.81	28.3	34[s81]	e

9	VC	s71	191.06	290.87	0.66	23.4	29[s103]	c
10	ZrC	s76	169.57	223.33	0.76	26.2	25.8[s104]	e
		s76	169.48	223.33	0.76	26.2		e
		s9	169.73	223.13	0.764	26.3		e
		s76	182.51	228.33	0.80	29.4		c
		s76	185.88	228.00	0.82	30.5		c
11	TiC	s73	198.27	286.00	0.69	25.8	24.7[s103]	c
		s73	176.90	250.33	0.71	24.5		c
		s9	182.18	242.00	0.75	27.1		e
		s73	187.80	241.67	0.78	28.8		e
		s73	174.54	221.33	0.79	28.0		c
12	B_4C	s105	192.00	226.00	0.85	32.8	30[s86]	e
13	TiN	s30	187.15	318.33	0.59	19.9	23[s104]	e
		s53	207.92	326.33	0.64	23.8		c
		s30	207.04	313.33	0.66	24.9		c
		s64	183.20	281.97	0.65	22.5		e
		s30	209.18	312.00	0.67	25.5		c
		s53	205.78	294.60	0.70	26.7		c
14	NbC	s50	171.68	340.00	0.50	15.2	18[s103]	e
		s50	170.99	333.33	0.51	15.5		c
15	NbN	s39	155.86	292.00	0.53	15.4	17[s103]	e
16	HfN	s30	193.11	305.67	0.63	22.4	17[s104]	e
17	Al_2O_3	s106	161.0	240.00	0.67	21.5	20[s81]	c
		s106	160.00	259.00	0.62	19.2		c
		s107	164.00	254.00	0.65	20.7		e
		s86	162.00	246.00	0.66	21.1		e
18	AlN	s106	134.7	206.00	0.65	18.4	18[s81]	c
		s108	130.2	212.10	0.61	16.5		c
		s109	123.3	207.50	0.59	15.2		c
		s110	132.00	211.10	0.63	17.0		e
		s86	128.00	203.00	0.63	16.9		e
19	GaN	s20	119.93	187.00	0.64	16.6	15.1[s81]	c
		s20	134.50	201.33	0.67	18.9		c
20	ZrO_2	s106	88.00	187.00	0.47	8.4	13[s81]	c
		s111	93.00	187.00	0.50	9.5		e
21	Si	s27	66.29	98.00	0.68	11.7	12[s81]	e
		s9	66.56	97.89	0.68	11.8		c
		s9	66.34	97.00	0.68	11.9		c
		s29	63.99	97.87	0.65	10.9		c
		s29	63.24	90.73	0.70	11.8		c
22	GaP	s18	54.76	87.73	0.62	9.0	9.5[s81]	c

		s8	56.09	88.60	0.63	9.4		e
		s9	55.77	88.75	0.63	9.24		e
		s18	55.65	88.19	0.63	9.3		e
		s18	61.89	89.67	0.69	11.5		c
23	AlP	s20	49.01	86.00	0.57	7.0	9.4[s81]	e
24	InN	s20	82.28	139.67	0.59	11.2	9[s103]	c
25	Ge	s9	54.35	74.94	0.73	11.2	8.8[s103]	e
		s8	54.80	75.00	0.73	11.4		e
		s29	53.14	72.20	0.74	11.3		c
		s29	43.78	60.33	0.73	9.5		c
26	GaAs	s18	45.82	76.80	0.60	7.2	7.5[s81]	c
		s8	46.49	75.00	0.62	7.8		e
		s9	46.67	75.40	0.62	7.8		e
		s18	46.74	75.53	0.62	7.8		e
		s18	49.29	76.33	0.65	8.7		c
27	Y_2O_3	s30	62.70	146.47	0.43	5.3	7.5[s81]	c
		s30	72.45	165.97	0.44	6.3		c
		s30	66.54	149.33	0.45	6.0		e
28	InP	s9	34.40	72.47	0.47	3.6	5.4[s81]	e
		s18	34.34	71.10	0.48	3.8		e
		s18	38.11	66.87	0.57	5.7		c
29	AlAs	s19	44.76	77.90	0.57	6.7	5[s81]	c
		s17	47.12	76.83	0.61	7.7		c
		s18	44.57	78.30	0.57	6.5		e
		s18	44.35	75.33	0.59	6.9		c
		s18	45.36	76.33	0.59	7.1		c
30	GaSb	s18	31.42	58.33	0.57	4.3	4.5[s103]	c
		s8	34.25	56.33	0.61	5.8		e
		s9	34.12	56.35	0.61	5.8		e
		s18	34.16	56.27	0.61	5.8		e
31	AlSb	s17	33.93	55.27	0.61	5.9	4[s81]	c
		s8	31.93	58.17	0.55	4.5		e
		s18	30.42	56.93	0.53	4.1		c
		s9	32.52	59.31	0.55	4.6		e
32	InAs	s8	29.49	59.07	0.50	3.4	3.8[s81]	e
		s9	29.50	57.94	0.51	3.6		e
		s18	32.38	59.60	0.54	4.5		c
33	InSb	s9	22.96	46.87	0.49	2.4	2.2[s81]	e
		s18	22.88	46.53	0.49	2.4		e
		s18	23.09	46.77	0.49	2.5		c
		s8	22.90	46.00	0.50	2.5		e

34	c-ZnS	s8	31.47	77.07	0.41	2.3	1.8[s103]	e
		s9	32.75	78.43	0.42	2.5		e
		s30	31.47	77.07	0.41	2.3		e
		s8	36.62	86.73	0.42	3.0		e
		s30	32.46	69.83	0.46	3.2		c
		s9	29.42	59.53	0.49	3.3		e
35	ZnSe	s8	28.76	63.13	0.46	2.7	1.4[s103]	e
36	ZnTe	s8	23.43	51.03	0.46	2.1	1[s103]	e
		s9	23.40	50.97	0.46	2.1		e

References:

[s1] Jahnatek, M.,Krajčí, M. &Hafner,J.Interatomic bonding, elastic properties, and ideal strength of transition metal aluminides: A case study for Al_3(V,Ti).Phys. Rev. B, **71**, 024101 (2005).

[s2]Adam,J. &Rich,J.B.The crystal structure of WAl_{12}, $MoAl_{12}$ and (Mn, Cr)Al_{12}.Acta Crystallogr.7, 813-816 (1954).

[s3]Barlock,J.G. & Mondolfo,L.F. Structure of some aluminium-iron-magnesium-manganese-silicon alloys. Zeitschrift fur Metallkunde.**66**, 605-611 (1975).

[s4]Darby,J.B. Jr., DowneyJ.W., NortonL.J., Intermediatephases in thetechnetium-aluminum and technetium-silicon systems, 8, 15-19 (1965).

[s5]Kirihara,K., Nakata, T., Takata, M.,Kubota,Y., Nishibori,E.,Kimura,K.& Sakata, M.Covalent Bonds in AlMnSi Icosahedral Quasicrystalline Approximant. Phys. Rev. Lett.85, 3468-3471 (2000).

[s6]Boer,F. R. &Pettifor,D. G. *Cohesion in Metals*(North-Holland, Amsterdam). 1 (1988).

[s7] Kamm, G. N. & Alers, G. A. Low-temperature elastic moduli of aluminum, J. Appl. Phys. 35, 327-330 (1964).

[s8] Every, A.G.&McCurdy,A.K.Landolt-Börnstein numerical data and functional relationships in science and technology, new series, group III: crystal and solid state physics. Low frequency properties of dielectric crystals, subvolume a: second and higher order elastic constants (Springer-Verlag, 1992), Vol. 29.

[s9] Frederikse,H.P.R. CRC Handbook of Chemistry and Physics.Elastic constants of single crystals. 87th edn (2006).

[s10] Wang,J., Shang,S.-L.,Wang,Y.,Mei,Z.-G.,Liang,Y.-F., Du,Y., &Liu,Z.-K. First-principles calculations of binary Al compounds: Enthalpies of formation and elastic properties. CALPHAD: Computer Coupling of Phase Diagrams and Thermochemistry. 35, 562-573 (2011).

[s11] Iotova,D., Kioussis,N.&Lim,S. P.Electronic structure and elastic properties of the Ni_3X

(X=Mn, Al, Ga, Si, Ge) intermetallics, Phys. Rev. B, 54, 14413 (1996).

[s12] Kim,D.E., Shang,S.L.&Liu,Z.K. Effects of alloying elements on elastic properties of Ni_3Al by first-principles calculations. Intermetallics. 18, 1163-1171 (2010).

[s13] Zhou,W., Liu,L. J., Li,B. L., Song,Q. G.& Wu,P. Structural, elastic and electronic properties of Al-Cu intermetallics from first-principles calculations, Journal of Electronic materials, 38, 356-64 (2009).

[s14] Guan, Y.Z., Zhang,H.Y.& Li,W. First-principles study on alloying stability, electronic structure, and mechanical properties of Al-based intermetallics, Physica B, 406, 1149-1153 (2011).

[s15] Hu,X.-L.,Zhang,Y.,Lu,G.-H.,Wang,T., Xiao,P.-H.,Yin,P.-G. &Xu, H. Effect of O impurity on structure and mechanical properties of NiAlintermetallics: A first-principles study. Intermetallics. 17, 358-364 (2009).

[s16] DavenportT., ZhouL., TrivisonnoJ., Ultrasonic and atomic force studies of the martensitic transformation induced by temperature and uniaxial stress in NiAl alloys,Phys. Rev. B, 59, 3421-3426 (1999).

[s17]Bouarissa, N.&Bachiri,R.Elastic constants and related properties of $Al_xGa_{1-x}As_ySb_{1-y}$/InAs. Physica B. 322, 193-200 (2002).

[s18] Azuhata,T., Sota,T.&Suzuki,K. Elastic constants of III-V compound semiconductors: modification of Keyes' relation. J. Phys.: Condens. Matter. 8, 3111-3119 (1996).

[s19] Gehrsitz,S., Sigg,H., Herres,N., Bachem,K., Kohler,K.&Reinhart,F. K. Compositional dependence of the elastic constants and the lattice parameter of $Al_xGa_{1-x}As$, Phys. Rev. B. 60, 11601-11610 (1999).

[s20] Marmalyuk, R., Akchurin,Kh.& Gorbylev,V. A. Evaluation of elastic constants of AlN, GaN, and InN. Inorganic Materials. 34, 691-694 (1998).

[s21]Ouyang,Y. F.,Liu,F. L.,Chen,H. M.,Tao, X. M., Du,Y. &He,Y. H. The structural stability,elastic constants and electronic structure of Al–Srintermetallics by first-principles calculations. Physica B. 406, 3681-3686 (2011).

[s22] Ghosh,G., Vaynman,S., Asta,M.& Fine,M. E. Stability and elastic properties of $L1_2$-(Al,

Cu)$_3$(Ti, Zr) phases: Ab initio calculations and experiments. Intermetallics. 15, 44-54 (2007).

[s23] Tao,X. M., Ouyang,Y. F., Liu,H. S., Zeng,F. J., Feng,Y. P., Du,Y.& Jin,Z. P. Ab initio calculation of the total energy and elastic properties of laves phase C15 Al_2RE (RE= Sc, Y, La-Lu).Computational Materials Science. 44, 392-399 (2008).

[s24]Tao,X. M., Ouyang,Y. F., Liu,H. S., Feng,Y. P., Du,Y.& Jin,Z. P. First-principles calculations of the thermodynamic and elastic properties of the $L1_2$-based Al_3RE (RE= Sc, Y, La-Lu). International Journal of Materials Research. 99, 582-588 (2008).

[s25] Hyland,R. W.and Stiffer,J. R. C. Scripta Metall. Mater., 25, 473-477 (1991).

[s26] Kamran,S.,Chen,K.Y.&Chen, L. Ab initio examination of ductility features of fcc metals. Physical Review B. 79, 024106 (2009).

[s27] Connétable,D.&Thomas,O. First-principles study of the structural, electronic, vibrational, and elastic properties of orthorhombic NiSi. Physical review B. 79, 094101 (2009).

[s28] Chen,K., Zhao,L. R.& Tse,J. S. Ab initio study of elastic properties of Ir and Ir_3X compounds. J. Appl. Phys. 93, 2414-2417 (2003).

[s29] Kang,K.& Cai,W. Brittle and ductile fracture of semiconductor nanowires-molecular dynamics simulations. Philosophical Magazine. 87, 2169-2189 (2007).

[s30] Yao, H.Z.,Ouyang, L.Z. &Ching, W.-Y. Ab initio calculation of elastic constants of ceramic crystals. J. Am. Ceram. Soc., 90, 3194-3204 (2007).

[s31] Hu,J.-Q., Xie,M., Pan,Y.,Yang,Y.-C.,Liu,M.-M.&Zhang,J.-M. The electronic, elastic and structural properties of Pd–Zr intermetallic.Computational Materials Science. 51,1-6 (2012).

[s32] Bannikov,V.V., Shein,I. R., and Ivanovskii,A. L. Trends instability, elastic and electronic properties of cubic Rh, Ir, Pd and Pt carbides depending on carbon content: MC versus M_4C from first-principles calculations. Journal of Physics and Chemistry of Solids.**71**, 803-809 (2010).

[s33] Zerarg,F., Bouhemadou,A., Khenata,R.& Binomran,S. FP-LAPW study of the structural,

elastic and thermodynamic properties of spinel oxides ZnX_2O_4 (X = Al, Ga, In). Computational Materials Science.50, 2651-2657 (2011).

[s34] Hou,Z.F. Effects of Cu, N, and Li intercalation on the structural stability and electronic structure of cubic Cu_3N. Solid State Sciences. 10, 1651-1657 (2008).

[s35] Bouhemadou, A. Elastic properties of mono- and polycrystalline $RCRh_3$ (R= Sc, Y, La and Lu) under pressure effect. Solid State Communications.149, 1658 (2009).

[s36]Kaur,N.,Mohan,R., Gaur,N.K.& Singh,R.K. The elastic and thermodynamic properties of antiperovskites: $MCNi_3$.Journal of Alloys and Compounds. 491, 284-290 (2010).

[s37] Peng,F.,Chen,D.,Fu,H.Z.&Cheng,X.L. Phase transition and elasticity of CdO under pressure, Phys. Status Solidi B. 246, 71–76 (2009).

[s38] Medkour,Y., Roumili,A., Maouche,D., Reffas,M. & Saoudi,A. First principles study of structural, elastic and electronic properties of ACY_3 (A = Al, In and Tl).Computational Materials Science. 47, 973-976 (2010).

[s39] Chen, X.-J.,Struzhkin, V. V., Wu, Z.G.,Somayazulu M., Jiang Q., Kung S., Chirstensen A. N., Zhao Y. S., Cohen R. E., Mao H-K., and Hemoley R. J., Hard superconducting nitrides, PNAS, 102, 3198-3201 (2005).

[s40] Ganeshan,S., Shang,S. L., Zhang, H., Wang,Y., Mantina,M.&Liu,Z. K. Elastic constants of binary Mg compounds from first-principles calculations. Intermetallics. 17, 313-318 (2009).

[s41] Lowther,J.E.Theoretical study of potential high-pressure phases of TaON and a quaternary $ZrTaO_3N$. Physical Review B. 73, 134110, (2006).

[s42] Bouhemadou,A., Zerarga,F., Almuhayya,A.& Bin-Omran,S. FP-LAPW study of the fundamental properties of the cubic spinel $CdAl_2O_4$. Materials Research Bulletin. 46,2252-2260 (2011).

[s43] Cherrad,D., Maouche,D., Louail,L.& Maamachea,M. Ab initio comparative study of the structural, elastic and electronic properties of $SnAMn_3$ (A = N, C) antiperovskite cubic compounds. Solid State Communications. 150, 782-787 (2010).

[s44] Meziani,A., Heciri,D.&Belkhir,H. Structural, electronic, elastic and optical properties

of fluoro-perovskite $KZnF_3$. Physica B. 406, 3646-3652 (2011).

[s45]Morris,J. R.,Ye,Y. Y.,Lee,Y.-B.,Harmon,B. N.,Gschneidner, K. A., &Russell,A. M. Ab initio calculation of bulk and defect properties of ductile rare-earth intermetallic compounds. ActaMaterialia. 52, 4849-4857 (2004).

[s46] Singh,R.P., Singh,R.K.& Rajagopalan,M. First-principle study on structural, elastic and electronic properties of rare-earth intermetallic compounds: TbCu and TbZn. Intermetallics. 19, 1359-1366 (2011).

[s47] Cherrad,D., Maoucheb,D., Reffas,M.,&Benamrania,A. Structural, elastic, electronic and optical properties of the cubic perovskites $CaXO_3$ (X = Hf and Sn). Solid State Communications. 150, 350-355 (2010).

[s48] Jiang,Y.,Smith,J. R.&Odette,G. R. Prediction of structural, electronic and elastic properties of $Y_2Ti_2O_7$ and Y_2TiO_5. ActaMaterialia. 58, 1536-1543 (2010).

[s49] Kadri,M. T., Derradji,N. E.& Belkhir,H. Elastic and electronic properties of fluorite-like boride Be_2B and its ternary compounds XBeB (X = Na, Mg, Al) from first principles. Phys. Status Solidi B. 247, 41-47 (2010).

[s50] Chen,J., Boyer,L. L., Krakauer,H.&Mehl,M. J. Elastic constants of NbC and MoN: Instability of B_1- MoN. Physical Review B. 37, 3295-3298 (1988).

[s51] Yang,F.,Wang,J.-W.,Ke,J.-L.,Pan,Z.-G.&Tang,B.-Y. Elastic properties and electronic structures of Mg–Ce intermetallic compounds from first-principles calculations, Phys. Status Solidi B. 248, 2097-2102 (2011).

[s52] Chen,K., Zhao,L. R., Tse,J. S.& Rodgers,J. R. Elastic properties of platinum Rh and Rh_3X compounds. Physics Letters A. 331, 400-403 (2004).

[s53] Chen,K., Zhao,L. R., Rodgers,J. R.& Tse,J. S. Alloying effects on elastic properties of TiN-based nitrides. J. Phys. D: Appl. Phys.**36,** 2725-2729 (2003).

[s54] Kanchana, V., Vaitheeswaran, G., Zhang, X. X.,Ma, Y. M., Svane, A., and Eriksson, O. Lattice dynamics and elastic properties of the 4f electron system: CeN. Physical Review B. 84, 205135 (2011).

[s55] Haddadi,K., Bouhemadou,A., Louail,L.&Maamache,M. Density functional study of the

structural, electronic, elastic and thermodynamic properties of ACRu3 (A = V, Nb and Ta) compounds. Intermetallics. 19, 476-485 (2011).

[s56] Peng,F.,Han,L. G.,Fu,H. Z.&Cheng,X. L. First-principles calculations on elasticity and the thermodynamic properties of TaC under pressure. Phys. Status Solidi B. 246, 1590-1596 (2009).

[s57] Mattesini,M., Ahuja,R.& Johansson,B. Cubic Hf_3N_4 and Zr_3N_4: A class of hard materials, Physical Review B, 68, 184108(2003).

[s58] Okamoto,N. L.,Nakano,T.,Tanaka,K.&Inui,H. Mechanical and thermal properties of single crystals of the type-I clathrate compounds $Ba_8Ga_{16}Ge_{30}$ and $Sr_8Ga_{16}Ge_{30}$. J. Appl. Phys. 104, 013529 (2008).

[s59] Gong,H. R. Ideal mechanical strengths of Ir and Ir_3Zr. ScriptaMaterialia. 59, 1197-1199 (2008).

[s60] Kanchana,V., Vaitheeswaran,G.,Ma,Y. M.,Xie,Y., Svane,A.& Eriksson,O. Density functional study of elastic and vibrational properties of the Heusler-type alloys Fe_2VAl and Fe_2VGa. Physical Review B. 80, 125108 (2009).

[s61] Hao,A. M.,Yang,X. C.,Wang,X. Y.,Yu,R. M.,Liu,X.,Xin,W. &Liu,R. P. First-principles investigations on electronic and elastic properties of YX (X = N, P, As and Sb) under high pressure.Computational Materials Science. 48, 59-64 (2010).

[s62] Coban,C.,Colakoglu,K. &Ciftci,Y. O. The structural, electronic, elastic, vibrational, and thermodynamic properties of HoX(X = Sb, Bi). Physica B. 405, 3977-3985 (2010).

[s63] Hou,Z. F. Elasticity, electronic structure, and dielectric property of cubic SrHfO3 from first-principles. Phys. Status Solidi B. 246, 135-139 (2009).

[s64] Podgursky,V. Ab initio calculations of elastic properties of isotropic and oriented $Ti_{1-x}Al_xN$ hard coatings. J. Phys. D: Appl. Phys.. 40, 4021-4026 (2007).

[s65] Haddadi,K., Bouhemadou,A., Louail, L.&Medkour,Y. Structural, elastic and electronic properties of $XNCa_3$ (X = Ge, Sn and Pb) compounds. Solid State Communications. 149, 619-624 (2009).

[s66] Hichour,M., Rached,D., Rabah,M., Benalia,S., Khenata,R.&Semari,F. Structural and

elastic properties of antiperovskites $XNBa_3$ (X = As, Sb) under pressure effect. Physica B. 404, 4034-4038 (2009).

[s67] Jha,P. K.&Guptaa,S. K. First principles lattice dynamical study of the cubic antiperovskite compounds $AsNBa_3$ and $SbNBa_3$. Solid State Communications. 150, 1650-1655 (2010).

[s68] Bouhemadou,A.&Haddadi,K. Structural, elastic, electronic and thermal properties of the cubic perovskite-type BaSnO3. Solid State Sciences. 12, 630-636 (2010).

[s69] Cui,S. X.,Feng,W. X.,Hu,H.Q.,Feng,Z. B. &Wang,Y. X. High-pressure structural, electronic and optical properties of $KMgF_3$: A first-principles study.Journal of Alloys and Compounds. 484, 597-600 (2009).

[s70] Haddadi,K., Bouhemadou,A.& Louail, L.Ab initio investigation of the structural, elastic and electronic properties of the anti-perovskite $TlNCa_3$. Solid State Communications. 150, 932-937 (2010).

[s71] Liu,H. L.,Zhu,J. C.,Liu,Y. &Lai,Z. H. First-principles study on the mechanical properties of vanadium carbides VC and V_4C_3. Materials Letters. 62, 3084-3086 (2008).

[s72] Liu,N.-N.,Song, R.-B.&Du,D.-W. Elastic constants and thermodynamic properties of $Mg_2Si_xSn_{1-x}$ from first-principles calculations. Chinese Physics B. 18, 1974-1984 (2009).

[s73] Chen,K. Y. &Zhao,L. R. Elastic properties, thermal expansion coefficients and electronic structures of $Ti_{0.75}X_{0.25}C$ carbides. Journal of Physics and Chemistry of Solids. 68, 1805-1811 (2007).

[s74]Mattesini,M. Elastic properties and electrostructural correlations in ternary scandium-based cubic inverse perovskites: A first-principles study. Physical Review B. 79, 125122 (2009).

[s75] Medkour,Y., Roumili,A., Maouche,D.& Maamache,M. First-principles study of the structural, electronic, and magnetic properties of $InCCo_3$ and $InNCo_3$. Solid State Communications. 151, 1916-1919 (2011).

[s76] Fu,H. Z., Peng,W. M. &Gao,T. Structural and elastic properties of ZrC under high pressure. Materials Chemistry and Physics. 115, 789-794 (2009).

[s77] Haddad,K.,Bouhemadou,A., Louail,L., Rahal,F.& Maabed,S. Prediction study of the structural, elastic and electronic properties of $ANSr_3$ (A = As, Sb and Bi).Computational Materials Science. 46, 881-886 (2009).

[s78] Haddadi,K., Bouhemadou,A.&Louail,L. First-principles study of the structural, elastic and electronic properties of the anti-perovskites $SnBSc_3$ and $PbBSc_3$.Journal of Alloys and Compounds. 504, 296-302 (2010).

[s79] Bouhemadou,A., Al-Douri,Y., KhenataR.& Haddadi,K. Structural, elastic, electronic, optical and thermal properties of *c*-$SiGe_2N_4$. Eur. Phys. J. B.**71**, 185-194 (2009).

[s80] Haddadi,K., Bouhemadou,A., Louail,L., Maabed,S.& Maouche,D. Structural and elastic properties under pressure effect of the cubic antiperovskite compounds $ANCa_3$ (A = P, As, Sb, and Bi). Physics Letters A. 373, 1777-1781 (2009).

[s81] Gao, F. M., He, J. L., Wu, E. D., Liu, S. M., Yu, D. L, Li, D. C., Zhang, S. Y., and Tian, Y. J., Hardness of covalent crystals. Physical Review Letters, 91, 015502(2003).

[s82]Zhao, Y., He, D. W.,Daemen, L. L.,Shen, T. D.,Schwarz, R. B., Zhu, Y., Bish, D. L.,Huang, J.,Shen, G., Qian, J., and Zerda, T. W.,Superhard B-C-N materials synthesized in nanostructured bulks. J.Mater. Res. 17, 3139-3145 (2002).

[s83] Solozhenko, V. L., Kurakevych, O. O., Andrault, D., LeGodec, Y., andMezouar, M., Ultimate Metastable Solubility of Boron in Diamond: Synthesis of Superhard Diamondlike BC5. Phys. Rev. Lett. 102, 015506 (2009).

[s84]Andrievski,R. A.,Superhard materials based on nanostructured high-melting point compounds: achievements and perspectives. Int. J. Refract. Met. Hard. Mater. 19, 447-452 (2001).

[s85] Chang,J., Chen. X. R., Wei, D. R., and Yuan, X. L., Elastic constants and anisotropy of β-BC_2Nunder pressure. Physica B: Condensed Matter. 405 (17), 3751-3755 (2010).

[s86] Teter,D. M., Computational alchemy: The search for new superhard materials. MRS Bulletin. 23 (1), 22-27 (1998).

[s87] Solozhenko,V. L., Andrault, D., Fiquet,G., Mezouar,M., and Rubie,D. C., Synthesis of superhard cubic BC_2N. Appl. Phys. Lett. 78, 1385-1387 (2001).

[s88] Wang,Y. J., and Wang, C. Y., Mechanical properties and electronic structure of superhard diamondlike BC_5 : A first-priciples study.Journal of Applied Physics. 106 (4), 043513 (2009).

[s89] Grimsditch,M.,Zouboulis,E. S., and Polian, A., Elastic constants of boron nitride.J. Appl. Phys. 76,832-834 (1994).

[s90] Westbrook,J. H., and Conrol, H., The Science of Hardness Testingand its Research Applications (ASM, Metals Park, OH, 1973).

[s91] Jiang, C.,Lin, Z. J., Zhang, J. Z., and Zhao, Y. S., First-principles prediction of mechanical properties of gamma-boron.Applied Physics Letters. 94(19), 191906 (2009).

[s92] Solozhenko,V. L., Kurakevych,O. O., and Oganow,A. R.,On the hardness of a new boron phase. J.Superhard Mater. 30, 428-429 (2008); Oganov,A. R., Chen,J. H., Gatti,C., Ma,Y. Z., Ma,Y. M., Glass,C. W., Liu,Z. X., Yu,T., Kurakevych,O. O., and Solozhenko,V. L.,Ionic high-pressure form of elemental boron.Nature (London) 457,863-867 (2009).

[s93]Zarechnaya, E. Y., Dubrovinsky, L.,Dubrovinskaia,N., Filinchuk,Y., Chernyshov,D., Dmitriev,V., Miyajima,N., Goresy,A.E., Braun,H. F.,et. al.Superhard Semiconducting Optically Transparent High Pressure Phase of Boron. Phys. Rev. Lett. 102, 185501 (2009).

[s94] Levine, J. B., Tolbert, S. H., and Kaner,R. B., Advancements in the Search for Superhard Ultra-Incompressible Metal Borides. Advanced Functional Materials. 19 (22), 3519-3533 (2009).

[s95]Chung,H. Y., Weinberger,M. B., Levine,J. B., Kavner,A., Yang,J. M., Tolbert,S. H., and Kaner,R. B.,Synthesis of Ultra-Incompressible Superhard Rhenium Diboride at Ambient Pressure,Science 316, 436-439 (2007).

[s96]Gu,Q. F., Krauss,G., and Steurer,W.,Transition Metal Borides: Superhard versus

Ultra-incompressible.Adv. Mater. 20, 3620-3626(2008).

[s97] Hao,X. F., Wu,Z. J., Xu, Y. H., Zhou,D. F., Liu,X. J., and Meng,J., Trends in elasticity and electronic structure of 5d transition metal diborides: first-principles calculations. J. Phys.: Condens. Matter 19, 196212 (2007); Koehler, M. R., Keppens, V., Sales, B. C., Jin, R. L., and Mandrus, D., Elastic moduli of superhard rhenium diboride.J. Phys. D: Appl. Phys. 42, 095414 (2009).

[s98] Zhou W., Wu H., and Yildirim T.,Electronic, dynamical, and thermal properties of ultra-incompressible superhard rheniumdiboride: A combined first-principles and neutron scattering study. Phys. Rev. B. 76, 184113(2007).

[s99] Levine,J. B., Nguyen, S. L., Rasool, H. I., Wright,J. A., Brown,S. E., and Kaner,R. B.,Preparation and properties of metallic, superhard rhenium diboride crystals, J. Am. Chem. Soc. 130, 16953-16958 (2008).

[s100] Dubrovinskaia, N., Dubrovinsky, L., and Solozhenko, V. L.,Comment on "Synthesis of Ultra-Incompressible Superhard Rhenium Diboride at Ambient Pressure". Science318, 1550c (2007).

[s101] Chung,H.-Y., Weinberger,M. B., Yang,J.-M., Tolbert,S. H., andKaner, R. B., Correlation between hardness and elastic moduli of the ultraincompressible transition metal diborides RuB_2, OsB_2, and ReB_2. Appl. Phys. Lett. 92, 261904 (2008).

[s102] Qin, J. Q., He,D. W., Wang,J. H., Fang,L., Lei,L., Li,Y. J., Hu,J.,Kou,Z., and Bi, Y., Is rhenium diboride a superhard material?Adv. Mater. 20, 4780-4783 (2008).

[s103]Simunek, A., Vackar, J.,Hardness of Covalent and Ionic Crystals: First-Principle Calculations. Phys. Rev. Lett. 96, 085501 (2006).

[s104] Gou, H.Y., Hou, L., Zhang, J.W., Gao, F.M.,Pressure-induced incompressibility of ReC and effect of metallic bonding on its hardness.Appl. Phys. Lett.,92, 241901 (2008).

[s105] Mcclellan,K. J., Chu,F., Roper,J. M.,Shindo,I.,Room temperature single crystal elastic constants of boron carbide.Journal of Materials Science. 36, 3403-3407(2001).

[s106] Iuga, M., Steinle-Neumann, G., and Meinhardt, J., Ab-initio simulation of elastic

constants for some ceramic materials.The European Physical Journal B. 58(2), 127-133 (2007).

[s107] Goto, T., Anderson,O. L., Ohno,I., and Yamamoto,S., Elastic constants of corundum up to 1825K.Journal of Geophysical Research-Solid Earth and Planets. 94(B6), 7588-7602 (1989).

[s108] Kanoun,M. B., Merad,A. E., Merad,G., Cibert,J., and Aourag,H., Prediction study of elastic properties under pressure effect for zincblende BN, AlN, GaN and InN.Solid-State Electron. 48 (9), 1601-1606 (2004).

[s109] Wright,A. F., Elastic properties of zinc-blende and wurtzite AlN, GaN, and InN.Journal of Applied Physics. 82(6), 2833-2839 (1997).

[s110] McNeil,L. E., Grimsditch,M., and French,R. H.,Vibrational spectroscopy of aluminum nitride. J. Am. Ceram. Soc. 76 (5), 1132-1136 (1993).

[s111] Milman,V., and Warren, M. C., Elastic properties of TiB_2 and MgB_2.Journal of Physics-Condensed Matter. 13(24), 5585-5595 (2001).

[s112] Tao, X. M., Liu, Y. Z., Wang, R. C., Ouyang, Y. F., Du, Y., He, Y. H., First-principles investigations of elastic, electronic and thermodynamic properties of $Al_{12}X$ (X= Mo, W and Re). Intermetallics. 24, 15-21 (2012).
